# Essential oils of *Bursera morelensis* and *Lippia graveolens* for the development of a new biopesticides in postharvest control

**DOI:** 10.1038/s41598-021-99773-0

**Published:** 2021-10-11

**Authors:** Yoli Mariana Medina-Romero, Ana Bertha Hernandez-Hernandez, Marco Aurelio Rodriguez-Monroy, María Margarita Canales-Martínez

**Affiliations:** 1 Laboratorio de Farmacognosia, Unidad de Biología, Tecnología y Prototipos (UBIPRO), Facultad de Estudios Superiores Iztacala, Universidad Nacional Autónoma de México, Av. de los Barrios No. 1, Los Reyes Iztacala, C.P. 54090 Tlalnepantla, Edo. de México Mexico; 2 Laboratorio de Investigación Biomédica en Productos Naturales, Carrera de Medicina, Facultad de Estudios Superiores Iztacala, Universidad Nacional Autónoma de México, Avenida de los Barrios Numero 1, Colonia Los Reyes Iztacala, Tlalnepantla, Edo. de México C.P. 54090 México

**Keywords:** Microbiology, Plant sciences

## Abstract

Fruit and vegetable crops that are not consumed immediately, unlike other agricultural products, require economic and time investments until they reach the final consumers. Synthetic agrochemicals are used to maintain and prolong the storage life of crops and avoid losses caused by phytopathogenic microorganisms. However, the excessive use of synthetic agrochemicals creates health problems and contributes to environmental pollution. To avoid these problems, less toxic and environment-friendly alternatives are sought. One of these alternatives is the application of biopesticides. However, few biopesticides are currently used. In this study, the biopesticide activity of *Bursera morelensis* and *Lippia graveolens* essential oils was evaluated. Their antifungal activity has been verified in an in vitro model, and chemical composition has been determined using gas chromatography-mass spectrometry. Their antifungal activity was corroborated in vitro, and their activity as biopesticides was subsequently evaluated in a plant model. In addition, the persistence of these essential oils on the surface of the plant model was determined. Results suggest that both essential oils are promising candidates for producing biopesticides. This is the first study showing that *B. morelensis* and *L. graveolens* essential oils work by inhibiting mycelial growth and spore germination and are environment-friendly biopesticides.

## Introduction

Crops are at risk of acquiring diseases due to postharvest pathogens that invade tissues (mainly through wounds at different stages)^1^. Among postharvest pathogens, fungi of the genus *Fusarium* cause diseases in different plants (including crop plants, fruits, and vegetables, among others), causing substantial financial losses in agriculture. Many plants have at least one *Fusarium*-associated disease in their lifetime. Diseases are diverse and vary in severity. For example, fruit or seed rot produces many types of mycotoxins, which unfavorably affect human and animal health^2^.

Usually, synthetic agrochemicals are used to maintain the health of fruits and vegetables after harvest, during transportation, and storage. These agrochemicals work by inhibiting mycelial growth and tissue colonization, as well as producing antioxidant enzymes and exopolysaccharides^3^. However, synthetic agrochemicals cause problems such as environmental degradation, loss of biodiversity, soil and water contamination, and adverse effects on health. Therefore, it is necessary to develop natural products with a similar biological activity^3–6^. Novel products (which are sufficiently active against the target organism) are used to protect crops by mitigating the adverse effects caused by phytopathogens. These can also be developed on a large scale by a relatively quick and easy process to be non-toxic and biologically selective^5,7^. The efficacy of alternative pesticides depends on many factors such as the crop species, season, pest complexity, cost of producing the control agent, geographic location, environmental regulations regarding registration and use, and the economic status of the country where it will be used^7^. Biopesticides are among these novel products.

Biopesticides are bioactive compounds that are metabolites of restricted distribution in the organism, produced by plants, animals, bacteria, and fungi, among other organisms, with potential use as pest control agents^8,9^. Biopesticides act by inhibiting or killing plant pathogens in closed areas, require minimal manipulation, are stable, and do not affect the environment^8–10^. However, only a limited number of natural products are used directly as active ingredients in crop protection representing only 3.26% of the global pesticide market. It is estimated that these products currently have a 20% market share penetration^5,7–9^.

The essential oils of the species *B. morelensis* and *L. graveolens* possess in vitro antifungal activity^11–13^, making them potential candidates for the development of biopesticides. Because essential oils are volatile, they are extracted by steam entrainment. Essential oils disintegrate rapidly and are degraded by various abiotic factors (making their handling easy since there are no residue problems). Since they are less persistent, they are safe for humans and the environment, have a broad spectrum of activity, and are easy to process and use^14^. The composition of essential oils of the same plant species varies depending on the physiological development and the plant’s degree of maturity, soil, and climatic conditions, among other factors^15^. Essential oils are primarily terpenoids and may also be acyclic monoterpene alcohols, monocyclic alcohols, aliphatic aldehydes, aromatic phenols, bicyclic alcohols, monocyclic ketones, monoterpene bicyclic ketones, acids, and esters^16^.

The purpose of this study was to develop a new biopesticide from the essential oils of *B. morelensis* and *L. graveolens* by determining their in vitro and in vivo efficacy in the post-harvest protection of cherry tomatoes infected with *Fusarium*.

## Results

### Chemical analysis of essential oils

The essential oil of *B. morelensis* was colorless (density: 0.87 g/mL), and that of *L. graveolens* was yellow with a density of 0.93 g/mL; the compositions of the essential oils are shown in Tables 1 and 2, respectively.

**Table 1:** Compounds present in the essential oil of *Bursera morelensis* identified by gas chromatography-mass spectrometry.

Compound	RT	KRI_e_	KRI_l_	Similarity (%)	Abundance (%)
Sabinene	3.718	567.42	931	94	1.24
α-Pinene	3.818	606.78	943	97	5.50
Camphene	3.977	665.12	1030	96	0.14
β-Phellandrene	4.212	743.26	968	91	5.31
β-Thujene	4.228	748.28	970	91	38.02
β-Pinene	4.344	783.59	997	87	4.47
α-Phellandrene	4.535	838.09	1008	91	29.97
α-Terpinene	4.654	870.01	1011	97	0.29
*p*-Cymene	4.738	891.69	1047	97	2.45
ϒ-Terpinene	5.051	967.00	1078	96	0.47
(±)-α-Terpinyl acetate	5.329	1027.67	1050	96	0.65
(+)-Trans-4-thujanol	5.639	1089.62	1161	87	0.05
(±)-4-Terpineol	6.132	1178.02	1424	93	0.30
β-Caryophyllene	8.026	1440.55	1456	99	9.90
α-Caryophyllene	8.243	1465.16	1480	97	0.88
Germacrene D	8.415	1484.07	1384	97	0.26
(−)-β-Cubebene	8.417	1484.29	931	96	0.12

*RT* retention time, *KRIe* Kovats Retention Index estimated (column used: 30 m × 0.25 mm × 0.25 µm; dead-time 2.0 min), *KRIl* Kovats Retention Index from literature (column used: 60 m × 0.22 mm × 0.25 µm). The KRIe values are different from those in the literature, but the compounds detected have the same mass spectrum, this information (mass spectra) is included in a Supplementary Information file.

**Table 2:** Compounds present in the essential oil of *Lippia graveolens* identified by gas chromatography-mass spectrometry.

Compound	RT	KRI_e_	KRI_l_	Similarity (%)	Abundance (%)
Ethyl 2-methylbutanoate	2.912	126.81	829	95	0.03
Sabinene	3.726	570.65	873	94	2.05
α-Thujene	3.734	573.87	928	91	2.17
(±)-α-Pinene	3.824	609.08	931	97	1.27
β-Thujene	3.916	643.31	968	90	0.06
Camphene	3.979	665.82	943	97	1.04
(±)-β-Pinene	4.274	762.50	961.7	97	0.41
β-Pinene	4.354	786.55	970	90	3.37
α-Phellandrene	4.529	836.44	997	91	0.69
(±)-3-Carene	4.582	850.88	1005	96	0.38
(±)-α-Terpinyl acetate	4.652	869.49	1078	96	3.66
*p*-Cymene	4.733	890.42	1011	95	13.78
(±)-Eucalyptol	4.821	912.47	1023	98	2.18
Ocimene	4.914	935.04	993	97	0.28
α-Terpinene	5.048	966.32	1008	97	11.95
*Cis*-(±)-4-thujanol	5.136	986.12	1068	96	0.43
2-Carene	5.336	1029.13	948	92	0.54
(±)-Linalool	5.398	1041.95	1081	93	0.97
(±)-Camphor	5.890	1136.03	1122	98	0.73
(+)-Borneol	6.058	1165.45	1088	97	1.03
(±)-4-Terpineol	6.143	1179.87	1161	93	2.00
(±)-α-Terpineol	6.237	1195.48	1172	97	0.20
1-Isopropyl-2-methoxy-4-methylbenzene	6.549	1244.91	1215	95	2.90
Carvotanacetone	6.798	1281.99	1194.54	96	0.10
Carvacrol	6.925	1300.17	1278	94	25.19
*p*-Thymol	6.971	1306.64	1332.3	90	5.51
Thymol	6.989	1309.15	1266	90	11.27
(−)-β-Caryophyllene	8.027	1440.66	1424	99	3.70
α-Caryophyllene	8.251	1466.05	1456	98	1.90
ϒ-Muurolene	8.358	1477.86	1494	95	0.09
α-Eudesmol	9.492	1592.05	1643	98	0.12

*RT* retention time, *KRIe* Kovats Retention Index estimated (column used: 30 m × 0.25 mm × 0.25 µm; dead-time 2.0 min), *KRIl* Kovats Retention Index from literature (column used: 60 m × 0.22 mm × 0.25 µm). The KRIe values are different from those in the literature, but the compounds detected have the same mass spectrum, this information (mass spectra) is included in a Supplementary Information file.

A total of 17 compounds, primarily terpenes, were identified in *B. morelensis* essential oil. The most abundant compounds were β-thujene (38.02%), α-phellandrene (29.97%), β-caryophyllene (9.90%), α-pinene (5.50%), and β-phellandrene (5.31%) (Table 1).

Table 2 shows the compounds present in the essential oil of *L. graveolens*. Thirty-one compounds were identified, mainly terpenes and aromatic phenols. The most abundant compounds were carvacrol (25.19%), p-cymene (13.78%), α-terpinene (11.95%), thymol (11.27%), and p-thymol (5.51%) (CAS#: 3228-02-2).

The total compositions of *B. morelensis* and *L. graveolens* respectively included sabinene (1.24 and 2.05%), camphene (0.14 and 1.04%), β-thujene (38.04 and 0.06%), β-pinene (4.47 and 3.37%), α-phellandrene (29.97 and 0.69%), α-terpinene (0.29 and 11.95%), p-cymene (2.45 and 13.78%), (±)-α-terpinyl acetate (0.65 and 3.66%), (±)-4-terpineol (0.30 and 2.00%), β-caryophyllene (9.90 and 3.70%), and α-caryophyllene (0.88 and 1.90%).

### In vitro antifungal activity

**Figure 1:**
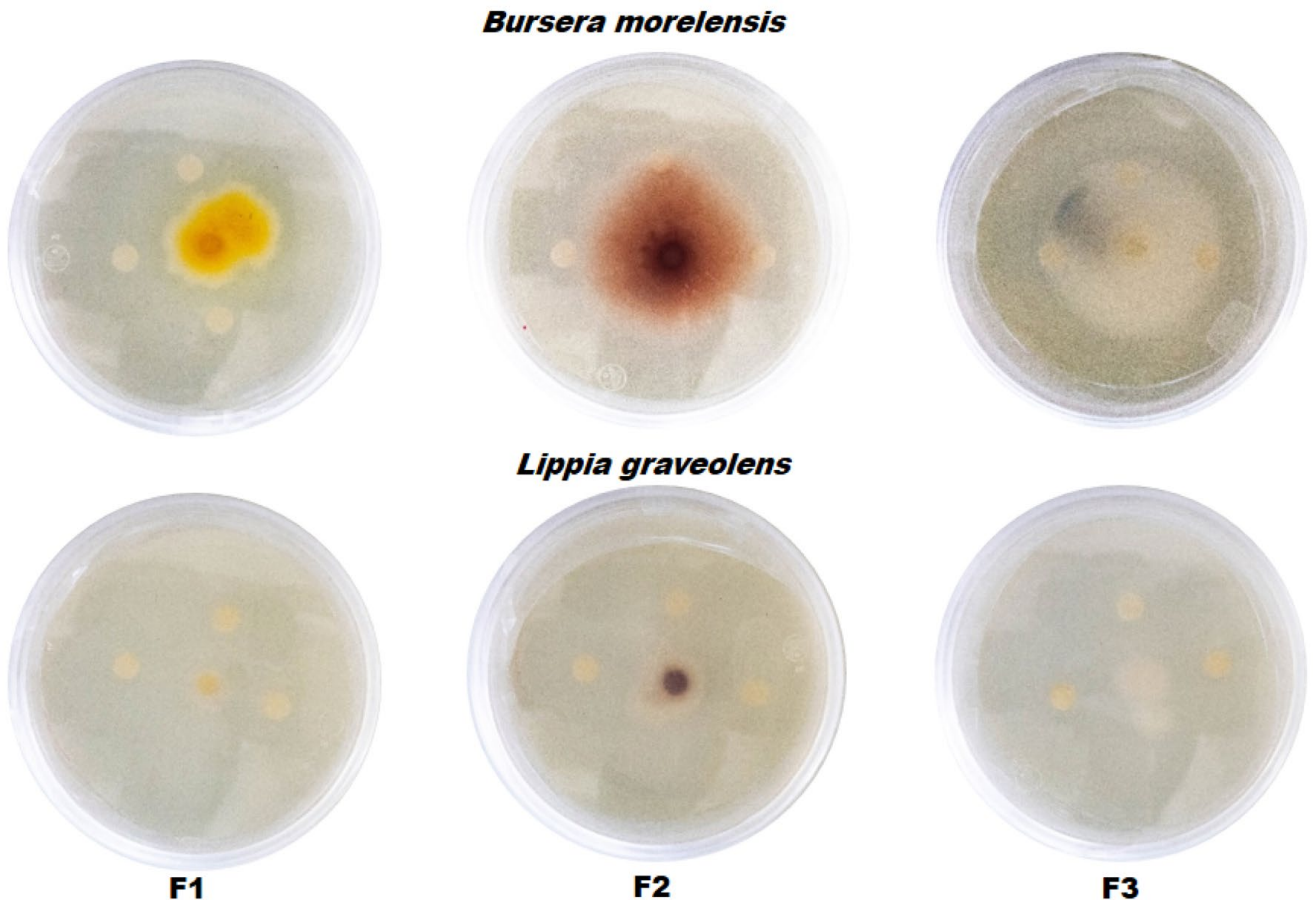
Effect of essential oils on the growth of three *Fusarium* strains in an in vitro model. Inhibition of radial growth of *Fusarium sporotrichioides* (F1), *Fusarium moniliforme* (F2), and *Fusarium solani* (F3) exposed to *Bursera morelensis* and *Lippia graveolens* essential oils.

**Figure 2:**
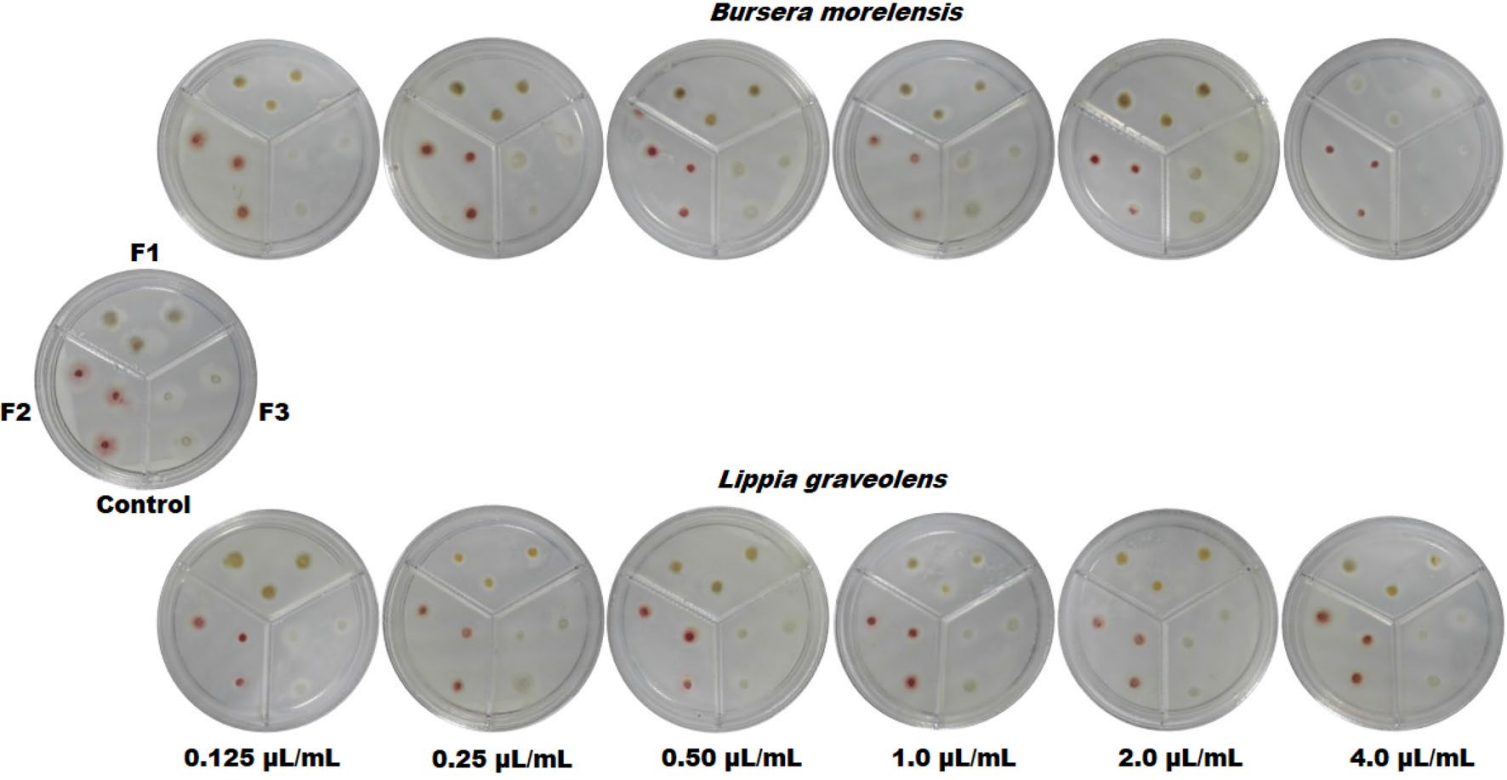
Effect of essential oils on the growth of three *Fusarium* strains in an in vitro model. Inhibition of radial growth of *Fusarium sporotrichioides* (F1), *Fusarium moniliforme* (F2), and *Fusarium solani* (F3) exposed to *Bursera morelensis* and *Lippia graveolens* essential oils at concentrations of 0.125, 0.25, 0.5, 1.0, 2.0 and 4.0 µL/mL.

The antifungal activity of *B. morelensis* and *L. graveolens* essential oils affected the mycelial growth of the three *Fusarium* species. The effects of the essential oils are shown in Figs. 1 and 2. Figure 1 shows the inhibition of *F. sporotrichioides*, *F. moniliforme*, and *F. solani* growth by the essential oils. In general, it was observed that the inhibition of the *Fusarium* species by *L. graveolens* oil was more significant than that caused by *B. morelensis* and that the most affected fungus was *F. sporotrichioides.*

**Figure 3:**
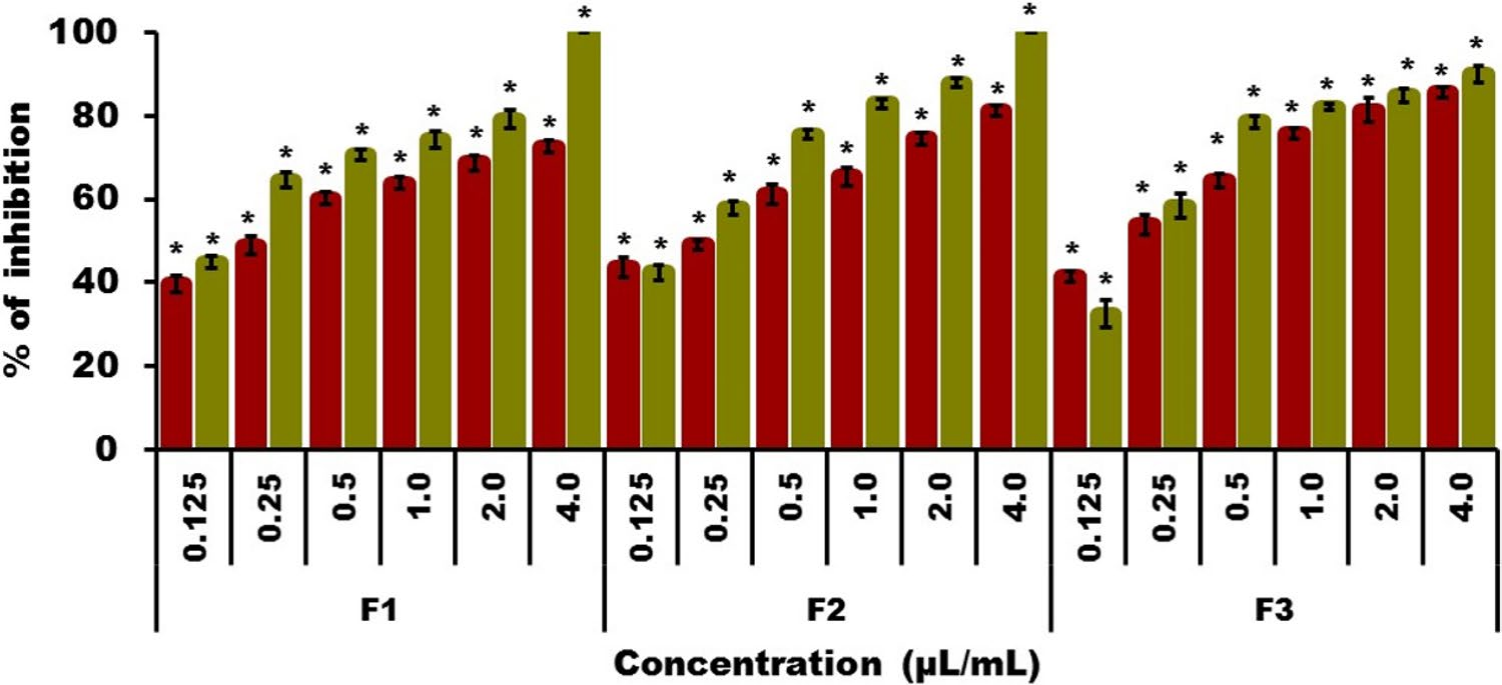
Effect of essential oils on the growth of three *Fusarium* strains. Percentage of growth inhibition in an in vitro model of *Fusarium sporotrichioides* (F1), *Fusarium moniliforme* (F2), and *Fusarium solani* (F3) exposed to *Bursera morelensis* (red) and *Lippia graveolens* (green) essential oils at concentrations of 0.125, 0.25, 0.5, 1.0, 2.0, and 4.0 µL/mL. *Statistically significant values of mean with respect to the control (P < 0.0001).

The mycelial growth inhibition of the three *Fusarium* species in an in vitro model by the essential oils is shown in Figs. 2 and 3, in addition to growth inhibition of *F. sporotrichioides*, *F. moniliforme*, and *F. solani*. A concentration-dependent inhibition was observed in the three *Fusarium* strains, equal to or higher than 60% inhibition at a concentration of 0.5 µL/mL. Higher growth inhibition was caused by *L. graveolens* essential oil (Fig. 3).

With regards to the *B. morelensis* essential oil activity against *F. sporotrichioides*, 60% growth inhibition was observed at a concentration of 0.5 µL/mL, and continued to increase as the concentration of oil increased, reaching 72% inhibition at a concentration of 4 µL/mL (which was the maximum concentration used in this test). When evaluating the *L. graveolens* essential oil, an inhibition greater than 60% was observed at a concentration of 0.5 µL/mL, which increased with the concentrations evaluated, until it reached a 100% inhibition at 4 µL/mL (Figs. 2 and 3). The mean inhibitory concentration (IC_50_) for *B. morelensis* essential oil was 0.27 µL/mL, and that for *L. graveolens* essential oil was 0.15 µL/mL.

*F. moniliforme* presented a growth inhibition higher than 60% when exposed to the essential oil of *B. morelensis* at a concentration of 0.5 µL/mL, increasing with essential oil concentration, until an inhibition of 81% at the maximum concentration evaluated (4.0 µL/mL). Exposure to *L. graveolens* essential oil produced an inhibition greater than 70% at a concentration of 0.5 µL/mL; the inhibition increased as the concentration increased, and a 100% inhibition was observed at the maximum concentration evaluated (Figs. 2 and 3). The mean inhibitory concentration (IC_50_) for *B. morelensis* essential oil was 0.31 µL/mL, and that for *L. graveolens* essential oil was 0.17 µL/mL.

The third fungus evaluated, *F. solani*, had an inhibition higher than 60% at a concentration of 0.5 µL/mL of *B. morelensis* oil; the inhibition increased with oil concentration, and at 4 µL/mL, it reached 85% inhibition. In evaluating *L. graveolens* oil, an inhibition higher than 75% was observed at a concentration of 0.5 µL/mL. At the maximum test concentration, an inhibition of 90% was observed (Figs. 2 and 3). The mean inhibitory concentration (IC_50_) for *B. morelensis* essential oil was 0.24 µL/mL and that for *L. graveolens* essential oil was 0.20 µL/mL.

*F. solani* was the most inhibited by *B. morelensis* essential oil (IC_50_ = 0.24 µL/mL), while *F. sporotrichioides* was the most inhibited by *L. graveolens* essential oil (IC_50_ = 0.15 µL/mL). However, the *L. graveolens* essential oil completely inhibited *F. sporotrichioides* and *F. moniliforme* at a concentration of 4 µL/mL.

### Antifungal activity in a plant model

**Figure 4:**
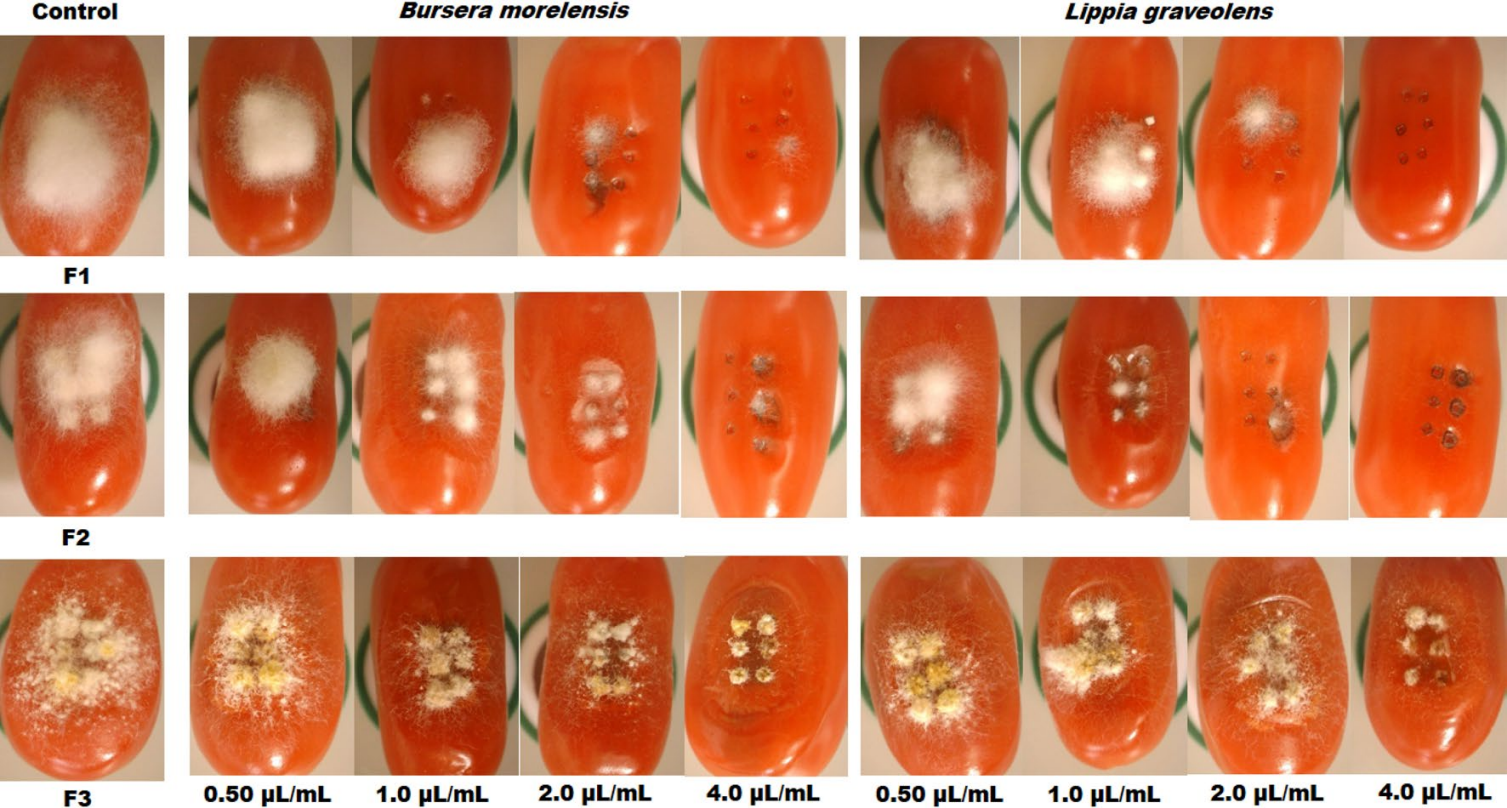
Effect of essential oils on the growth of three *Fusarium* strains in a plant model. Inhibition of *Fusarium sporotrichioides* (F1), *Fusarium moniliforme* (F2), and *Fusarium solani* (F3) growing on cherry tomatoes exposed to *Bursera morelensis* and *Lippia graveolens* essential oils at concentrations of 0.5, 1.0, 2.0, and 4.0 µL/mL.

**Figure 5:**
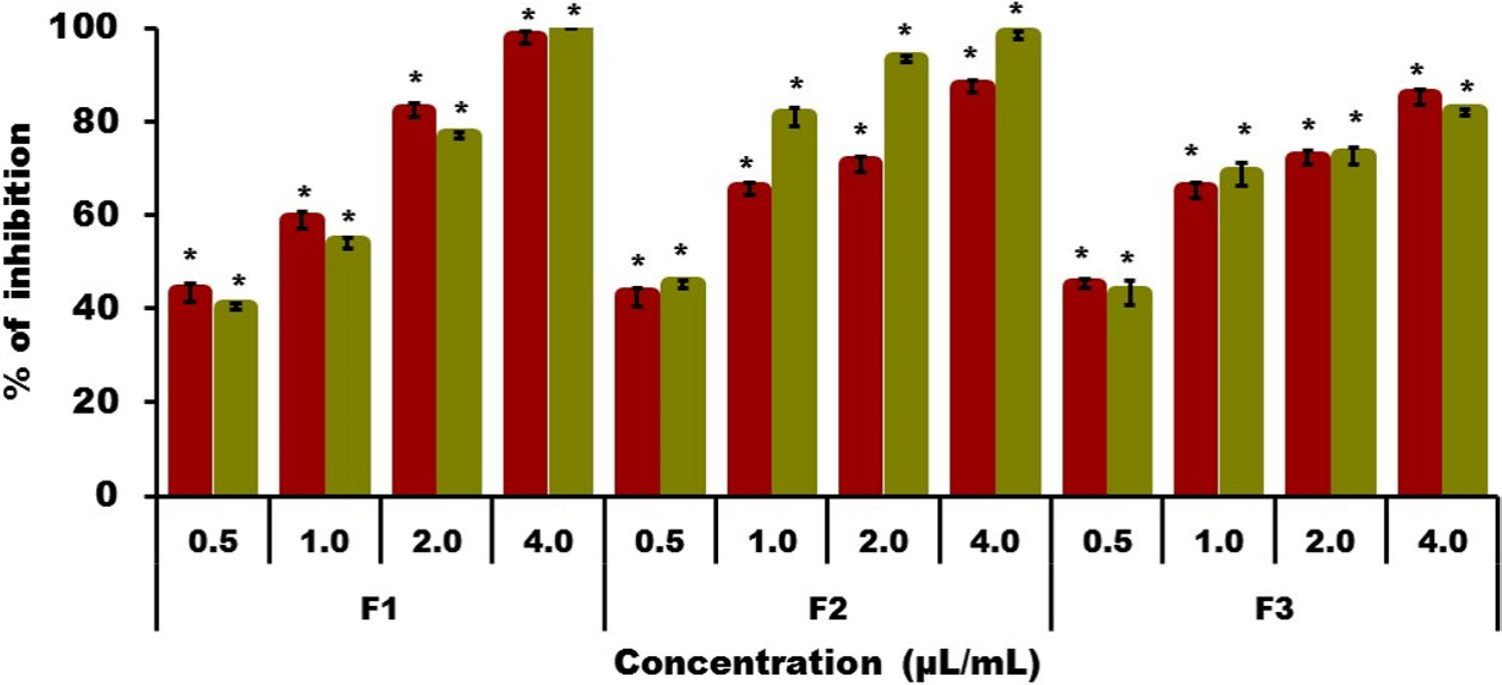
Effect of essential oils on the growth of three *Fusarium* strains. Percentage of inhibition of *Fusarium sporotrichioides* (F1), *Fusarium moniliforme* (F2), and *Fusarium solani* (F3) growing on cherry tomatoes exposed to *Bursera morelensis* (red) and *Lippia graveolens* (green) essential oils at concentrations of 0.5, 1.0, 2.0, and 4.0 µL/mL. *Statistically significant values of mean with respect to the control (P < 0.0001).

The effect of *B. morelensis* and *L. graveolens* essential oils on the conidia of the three *Fusarium* species growing on cherry tomatoes is shown in Figs. 4 and 5. Figure 4 shows the inhibition of fungal growth when exposed to the two essential oils. In general, concentration-dependent growth inhibition was observed for both the essential oils.

At the first three test concentrations of *B. morelensis* (0.5, 1.0, and 2.0 µL/mL), there was more significant growth inhibition of *F. sporotrichioides*. At the maximum test concentration (4.0 µL/mL), fungal growth inhibition by *B. morelensis* oil was 98%, while that by *L. graveolens* oil was 100% (Figs. 4 and 5). The mean inhibitory concentration (IC_50_) for *B. morelensis* oil was 0.66 µL/mL while that for *L. graveolens* oil was 0.76 µL/mL.

For the fungus *F. moniliforme*, the four test concentrations (0.5, 1.0, 2.0, and 4.0 µL/mL) of *L. graveolens* oil showed more significant growth inhibition than that by *B. morelensis* oil, and at the maximum test concentration, *B. morelensis* oil inhibited fungal growth by 87%, while *L. graveolens* oil inhibited it by 98% (Figs. 4 and 5). The mean inhibitory concentration (IC_50_) for *B. morelensis* oil was 0.63 µL/mL, while that for *L. graveolens* oil was 0.54 µL/mL.

Finally, the growth of *F. solani* was similarly inhibited by the four test concentrations (0.5, 1.0, 2.0, and 4.0 µL/mL) of *B. morelensis* and *L. graveolens* essential oils. In this case, the fungal growth inhibition was higher for *B. morelensis* (85%) than for *L. graveolens* (82%) (Figs. 4 and 5). The mean inhibitory concentration (IC_50_) for *B. morelensis* oil was 0.62 µL/mL, and that for *L. graveolens* oil was 0.62 µL/mL.

### Determination of persistence of essential oils

The compounds identified in tomato without essential oil were: nonadecane, 9-methyl (18.07%), eicosane (7.98%), and hentriacontane (73.96%). In the tomatoes treated with *B. morelensis* essential oil, the compounds found were: tetracontane (19.43%), eicosane (6.34%), and heneicosane (74.23%), whereas in tomato treated with *L. graveolens* essential oil, nonadecane, 9-methyl (17.22%), eicosane (8.54%), and hentriacontane (74.24%) were found. In the three treatments, eicosane was identified, and for the untreated tomatoes and those treated with *L. graveolens* essential oil, nonadecane, 9-methyl, and hentriacontane were identified.

With regards to untreated wounded tomatoes, we found hentriacontane (17.26%), eicosane (7.14%), and heneicosane (64.28%). In tomatoes treated with *B. morelensis* essential oil, no compounds could be identified, whereas, in tomatoes treated with the *L. graveolens* oil, we identified hentriacontane (15.59%), eicosane (7.62%), and docosane (57.42%). Hentriacontane and eicosane were identified in both treatments.

## Discussion

The results in this work agree with those of Rivera-Yañez et al. (2017)^13^ and Salas-Oropeza et al. (2020)^17^ on *B. morelensis* essential oil from the same collection site (San Rafael Coxcatlan), who reported the presence of the following compounds with their respective reported abundances: α-pinene (2.85 and 8.37%), α-phellandrene (1.58 and 0.80%), β-phellandrene (18.27 and 35.25%), α-caryophyllene (0.27 and 5.19%), and γ-terpinene (65.46 and 0.18%), among other compounds. However, it is essential to mention that they were not detected at the same concentrations. These differences may be due to various factors such as the season of collection^18^, different trees sampled, or changes in the physicochemical characteristics of both the soil and water^19^.

Hernández et al. (2008)^20^ studied the essential oil of L. graveolens collected in Zapotitlán de las Salinas and identified the following compounds: carvacrol (37.84%), thymol (6.72%), (±)-α-terpinyl acetate (22.35%), α-thujene (1.03%), α-caryophyllene (1.24%), β-pinene (2.54%), and (±)-linalool (0.26%). Similar to the comparisons of *B. morelensis* essential oil composition between different studies, *L. graveolens* essential oil composition and concentration differences might be due to differences in harvest time, the effects of light, and soil moisture^21^.

Carvacrol and thymol have been identified in previous studies on *B. morelensis* and *L. graveolens*^13,20,22^, as well as in other plants^23,24^. In these studies, their antimicrobial, antifungal, and antioxidant activities have been reported^13,20,22–24^. The diverse chemical composition and antimicrobial activity of *B. morelensis* and *L. graveolens* make them ideal candidates for biopesticide production in the postharvest control of fungal diseases.

The variations in the effect produced by the *B. morelensis* and *L. graveolens* essential oils on the three test microorganisms were on account of their differing compositions. It is probable that some of the compounds present in *L. graveolens* affect fungal growth to a greater extent and that these compounds also act synergistically^13,23^.

Inhibition of *F. sporotrichioides* mycelial growth by the essential oils may be due to the antifungal activity of these oils on account of their terpene compounds which disintegrate fungal hyphae^25^.

The mycelial growth inhibition of *F. moniliforme* by the essential oils may be due to compounds with antifungal activity present in the oils. Another possible mechanism is the increase in membrane permeability; the lipophilic properties of essential oils give them antifungal activity, as they are able to penetrate cell walls and affect the enzymes involved in cell wall synthesis^26^.

The mycelial growth inhibition of *F. solani* by the essential oils may be due to the inherent phenols, aldehydes, and alcohols, causing depolarization of mitochondrial membranes, which decreases the membrane potential, affecting ion channels, reducing the pH gradient, and affecting the proton pump and the ATP pool. As a result of all these processes, there is a change in membrane fluidity. Changes in fluidity cause membranes to become abnormally permeable, causing leakage of radicals, cytochrome *c*, calcium ions, and proteins as in the case of oxidative stress and bioenergetic failure^27^.

The antifungal activity of essential oils might be caused by the properties of terpenes which are highly lipophilic and have a low molecular weight, and can disrupt the cell membrane causing cell death or inhibiting mycelial growth and sporulation. Additionally, several in vitro studies have shown that terpenes do not have antimicrobial activity when tested separately; however, when the complete essential oil is used, significant antimicrobial activity is observed, and therefore there is a synergism between all the essential oil components^28,29^.

In addition to inhibiting mycelial growth, essential oils reduce or completely inhibit conidia production due to the compounds present in them, such as terpenes, alcohols, phenols, and ketones^26,30^. For example, to enter the cell wall, the hydroxyl groups interfere with membrane lipids and generate biochemical changes in the gradient, pH, and electrolytes. Due to these changes, the adaptation of fungi during the colonization of fruits and vegetables, particularly during the germination phase, is strongly affected^31^.

In a study conducted by Kadoglidou et al. (2011)^26^, it was shown that terpenes, particularly carvacrol, in addition to inhibiting mycelial growth, significantly inhibited the sporulation of the evaluated fungi through the strong suppression of conidia production. In addition, it was observed that inhibition was higher on sporulation than on mycelial growth.

The essential oils affect mycelial growth and conidial germination by decreasing the production of ergosterol, which is the principal sterol component of the fungal cell membrane, and plays a vital role in maintaining cell function and integrity. The decrease in ergosterol content leads to an imbalance in cell permeability and disruption of cell organelles. There is also a significant leakage of vital cell ions (Ca^2+^, K^+^, and Mg^2+^), deformation in conidiophores, and conspicuous depressions in conidia^32^.

Fungi of the genus *Fusarium* produce at least one disease in most crops. These fungi infect wounded or damaged fruits and vegetables, which facilitates infection and subsequent colonization. Therefore, this type of postharvest disease can be avoided by inhibiting mycelial growth and conidia production. The severity of the disease depends on the host plant and the diseased target organism, so it is difficult to find the best conditions in which to control these diseases, in addition to requiring investments of time and money for their treatment^2,33,34^. By using *B. morelensis* and *L. graveolens* essential oils, which inhibit mycelial growth and reduce or completely inhibit conidial germination^26,30^, protection and prevention effects have been observed^30^. The essential oils of *B. morelensis* and *L. graveolens* have a chemically varied composition, and several of their components, such as α-pinene and carvacrol, have been reported to have antifungal activity. These compounds have been demonstrated to destroy cell integrity, inhibit respiration and ion transport processes, and increase membrane permeability^13,23^.

In summary, *B. morelensis* and *L. graveolens* essential oils are likely to be successful in the postharvest control of *Fusarium* spp. In the present study, both essential oils are demonstrated to be biopesticide candidates since only a minimum amount of sample is required to have an inhibitory effect on the growth of the three fungi. The application of *B. morelensis* and *L. graveolens* essential oils during fruit and vegetable storage for controlling postharvest infections caused by *Fusarium* spp has two significant effects. First is protection, since it prevents the disease from becoming more severe by inhibiting mycelial growth, and second is prevention, since it inhibits conidial germination^30^. Additionally, essential oils are natural, safe, eco-friendly, cost-effective, renewable, and readily biodegradable antifungal agents for food preservation^35^.

Regarding the determination of the persistence of essential oils on the surface of tomatoes, the similarities between the compounds found in tomatoes indicate that essential oils do not remain for long on the fruit surface, demonstrating that these two essential oils have little persistence following application, making them good biopesticide candidates. Essential oils and their components, which are of natural origin, are quickly degraded in the environment or evaporate, which is why they are considered favorable for the environment^36,37^. To treat postharvest diseases, synthetic fungicides are more harmful because there is only a short period between treatment and consumption of the products^30^. Due to their low persistence, the use of essential oils can improve food safety by eliminating fungal spread and leaving no detectable residues after storage^38^.

## Methods

### Collect of plant material

The medicinal plant *B. morelensis* was collected in San Rafael, Coxcatlán, in the Tehuacán-Cuicatlán valley, between 18°12′ and 18°14′ N, and 97°07′ and 97°09′ W, at an altitude of 957–1400 m above sea level. The medicinal plant *L. graveolens* was collected in Zapotitlán de las Salinas, between 18°07′18″ N and 97°19′24″–97°30′06″ W, at an altitude of 1200–2500 m above sea level. Both species were collected in January 2020. Voucher specimens were deposited in the Iztacala herbarium (IZTA) (*B. morelensis* voucher IZTA 42123 and *L. graveolens* voucher MCM8). *B. morelensis* and *L. graveolens* species are not endangered. The specimens were collected in the field with permission from the “Secretaria de Medio Ambiente y Recursos Naturales” (SGPA/DGVS/1266). The study on these two medicinal plants species has comply with relevant institutional, national, and international guidelines and legislation".

### Obtention of essential oils

The branches of *B. morelensis* and *L. graveolens* were cut into 1–2-cm fragments using pruning shears. The essential oil was obtained using the hydrodistillation method where 400 g of the fresh plant was placed in 500 mL of distilled water in a 1000-mL ball flask with a heating mantle (SEV-Prendo, MC301-9, Mexico City, Mexico) connected to a double-pass condenser (designed by the Laboratory of Pharmacognosy at the FES Iztacala, UNAM, Tlalnepantla, Estado de México, Mexico). The condenser was attached to a cold-water circulator at a controlled temperature. Distillation proceeded for 40 min, and the process was repeated for all of the plant material. The essential oil was separated from the aqueous phase by density difference and stored in an amber glass vial at − 18 °C^13^.

### Chemical analysis of essential oils

The essential oils were analyzed by gas chromatography–mass spectrometry using a model 6850 chromatograph (Agilent Technologies, Santa, Clara, CA, USA) coupled with a model 5975C mass spectrometer (Agilent Technologies) and an HP-5MS column (30 m × 0.25 mm, 0.25 µm; Agilent Technologies). For the sample, 1 µL of essential oil was injected in split mode. The separation conditions were as follows: an initial temperature of 70 °C for 2 min followed by two increases in the heating ramps; the first at 20 °C min^−1^ to 230 °C, and the second at 8 °C min^−1^ up to 280 °C and then maintained for 1 min, using helium as a carrier gas. The total analysis time was 17.25 min. The detected mass range was 35–750 m/z, the sample was ionized by electronic impact at 70 eV, and the ionization source temperature was 230 °C. The essential oil components were identified by comparison with the NIST version 8.0 library database (National Institute of Standards and Technology, Gaithersburg, MD, USA) and the retention index^13^.

### In vitro antifungal activity

The strains *Fusarium sporotrichioides* ATTC NRLL3299, *Fusarium moniliforme* CDBB-H-265, and *Fusarium solani* CDBB-1407 were used.

Antifungal activity was determined using the radial growth inhibition method^39^. Petri dishes with 30 mL of potato dextrose agar (Bioxon, Estado de Mexico, Mexico) were used. A 5-mm diameter inoculum from each strain was placed in the center of each Petri dish. After mycelial growth, sterile paper filter discs were placed at 0.5 cm from the colony’s edge. Each disc was impregnated with a concentration of 5 µL of *B. morelensis* or *L. graveolens* essential oil. Ketoconazole was used as a positive control (disc with 7 µg). The Petri dishes were incubated at 23 °C for 72 h until the colony growth enveloped the control group discs, and crescents of inhibition formed around the discs containing the essential oil^39^. Bioassays were performed in triplicates.

The radial growth inhibition method was used to determine the medium fungicidal concentration (CF_50_). The concentrations of *B. morelensis* and *L. graveolens* essential oil used were: 0.125, 0.25, 0.5, 1.0, 2.0, and 4.0 µL/mL. Three 0.5-mm diameter inocula of the same strain were placed equidistantly in each compartment of a divided Petri dish. *B. morelensis* and *L. graveolens* essential oil at varying concentrations were sprayed in each compartment containing each of the three inocula of each strain and incubated at 23 °C for 72 h. Subsequently, the colony area was measured, and colony growth inhibition was determined^39^.

### Antifungal activity in a plant model

To evaluate the antifungal activity of *B. morelensis* and *L. graveolens* essential oils in an in vivo model, *Solanum lycopersicum* var*. cerasiforme* (cherry tomato) were obtained from a local market; woundless individuals of similar size and weight were selected. Three groups were used for the tomatoes: a control group (no wounds), a wound group, and a wounded group inoculated with the *Fusarium* strains. The tomatoes were washed under tap water and superficially sterilized with 75% ethanol for 30 s. Subsequently, they were rinsed thrice with sterile distilled water and dried in a laminar flow hood for 60 s. Once sterilized, six equidistant wounds 2 mm deep were made using a sterile dissection needle for the tomatoes with the wound treatment. The wounded tomatoes were inoculated with 60 µL of the spore suspension of any of the *Fusarium* strains. The conidial suspensions were prepared using sterile distilled water and 3.9 × 10^7^ conidia/mL of *F. sporotrichioides* ATTC NRLL3299, *F. moniliforme* CDBB-H-265, or *F. solani*. Each essential oil was suspended with sterile distilled water and sprayed on the tomatoes to test the antifungal activity (depending on the treatment), using the following concentrations: 0.5, 1.0, 2.0, and 4.0 µL/mL, to obtain the average fungicide concentration (CF_50_). The tomatoes were placed in sterile plastic containers at room temperature under a 12:12 h light/dark photoperiod for 8 days. A control group without essential oil treatment was used in all three treatments. After 8 days, the colony size was measured using the ImageJ program to determine the growth area of the mycelium^40,41^.

### Determination of persistence of essential oils

Persistence tests in sterile plastic containers were performed on the tomatoes. There were two experimental groups (n = 3 per group) for each essential oil: wounded and woundless tomato groups. Both groups were sprayed with the essential oils of *Bursera morelensis* or *Lippia graveolens* at the maximum concentration used in the previous experiments (4.0 µL/mL). The tomatoes, wounded and woundless, but without essential oil application, served as a control. The containers were placed at room temperature under a 12:12 h light/dark photoperiod for 8 days. Once the time had elapsed, the cherry tomatoes were placed in a flask with hexane and washed. Subsequently, the wash was injected into the GC–MS under the same conditions previously mentioned. The components present on the surface of the cherry tomatoes exposed to the biopesticides were analyzed using gas chromatography–mass spectrometry and compared with the control to observe possible differences between the treatments.

### Statistical analysis

The mean and standard deviation of the experiments were determined. Analysis of variance (ANOVA) was performed to test for significant differences (p < 0.05) with Tukey’s HSD multiple comparison test using the GraphPad Prism version 7 program. The IC_50_ values were determined by Probit analysis, based on the percentage of inhibition obtained for each concentration tested, using the same program^41,42^.

## Supplementary Information

Supplementary Information.

## Data Availability

The mass spectra is available in a Supplementary Information file.
