# Supplementary material for: Essential oils of Bursera morelensis and Lippia graveolens for the development of a new biopesticides in postharvest control
Source: Sci Rep. 2021 Oct 11;11:20135. doi: 10.1038/s41598-021-99773-0 (PMC8505479; doi:10.1038/s41598-021-99773-0)
Supplement: Supplementary file 1 — Supplementary Information. [file 41598_2021_99773_MOESM1_ESM.pdf]

MASS SPECTRA

Mass spectra that we obtained from each sample, the experimental mass spectra in part superior along with that of the literature, and finally the mass spectrum of the literature with the structure of the compound. The standards used appear first, then the 17 compounds identified in *Bursera morelensis* essential oil, and finally, the 31 compounds present in *Lippia graveolens* essential oil.

Standars

1-Octene

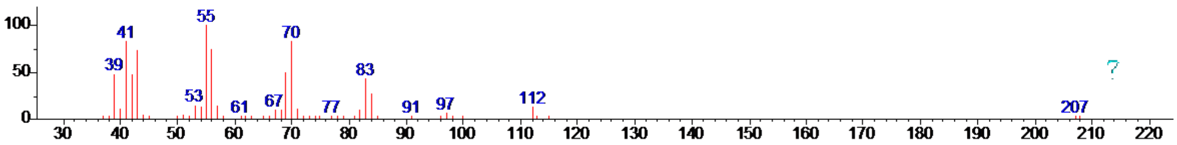

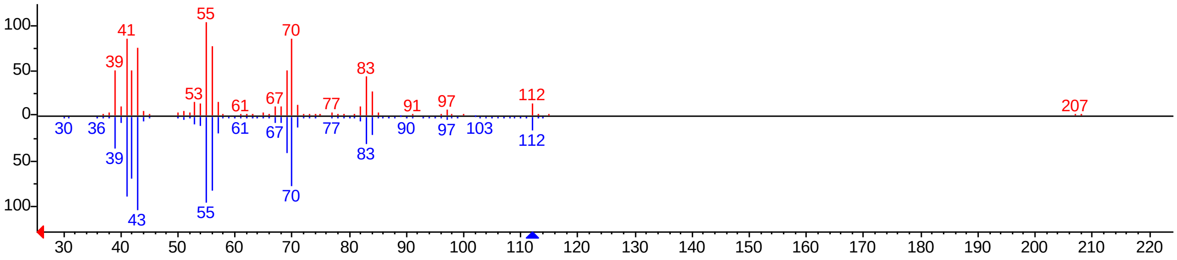

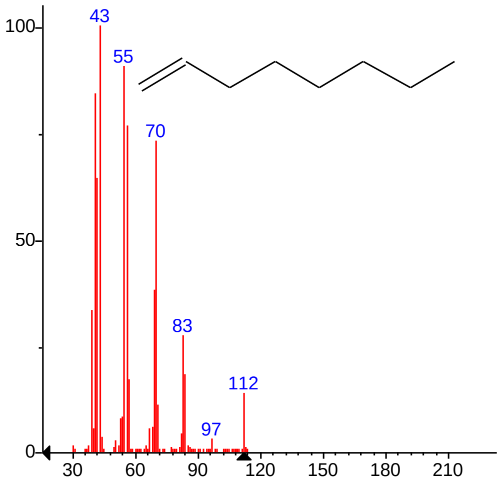

Octadecane

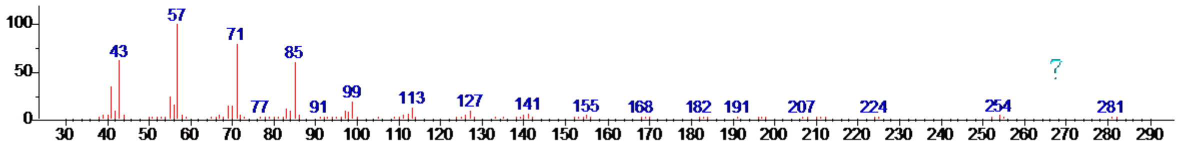

100
50
0
57
43
71
85
77
91
99
113
127
141
155
168
182
191
207
224
254
281
?
30 40 50 60 70 80 90 100 110 120 130 140 150 160 170 180 190 200 210 220 230 240 250 260 270 280 290

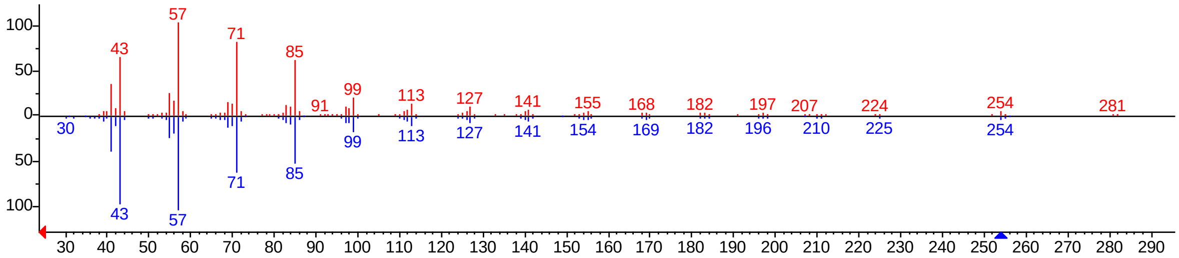

100
50
0
50
100
57
43
71
85
91
99
113
127
141
155
168
182
197
207
224
254
281
30
99
113
127
141
154
169
182
196
210
225
254
71
85
43
57
30 40 50 60 70 80 90 100 110 120 130 140 150 160 170 180 190 200 210 220 230 240 250 260 270 280 290

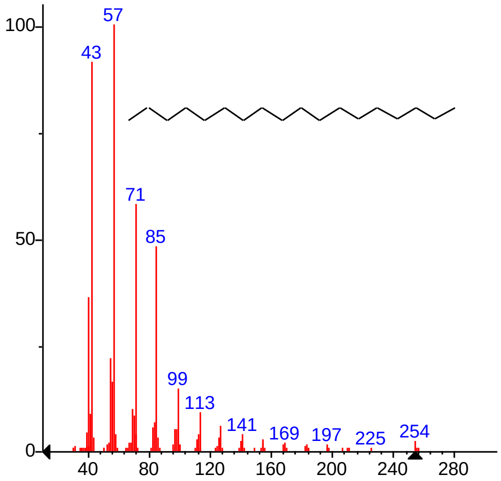

100
50
0
57
43
71
85
99
113
141
169
197
225
254
40 80 120 160 200 240 280

Bursera morelensis essential oil

Sabinene

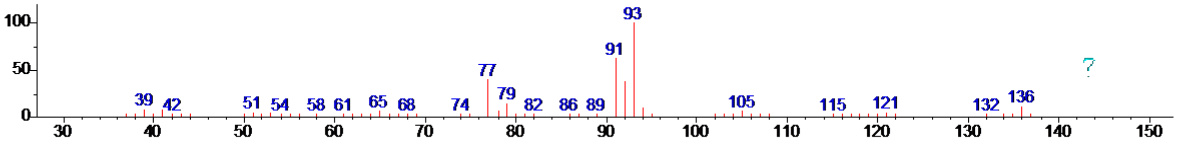

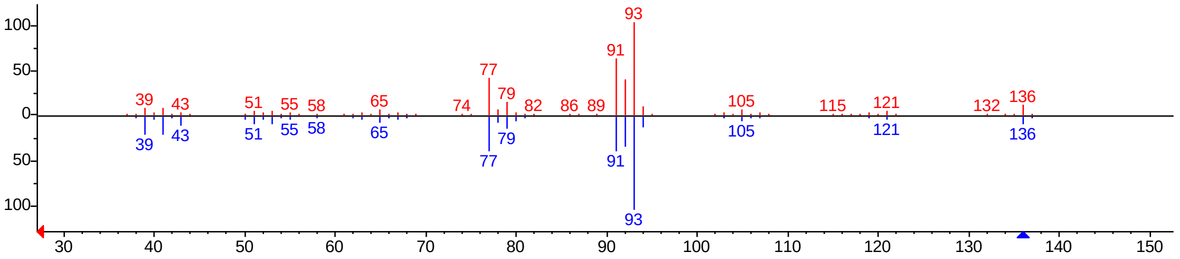

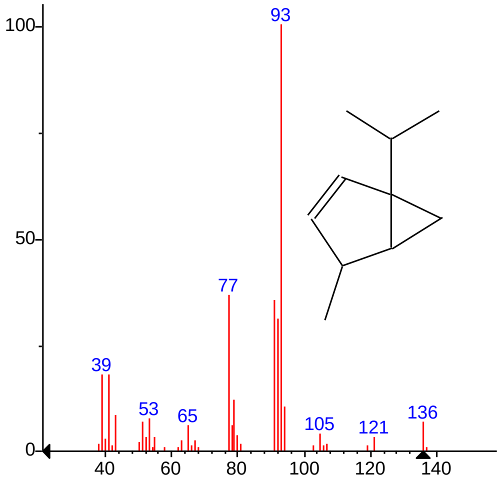

α-Pinene

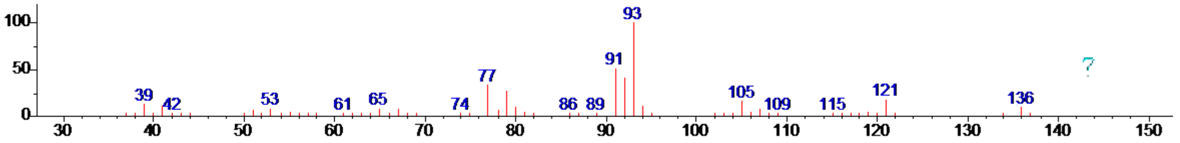

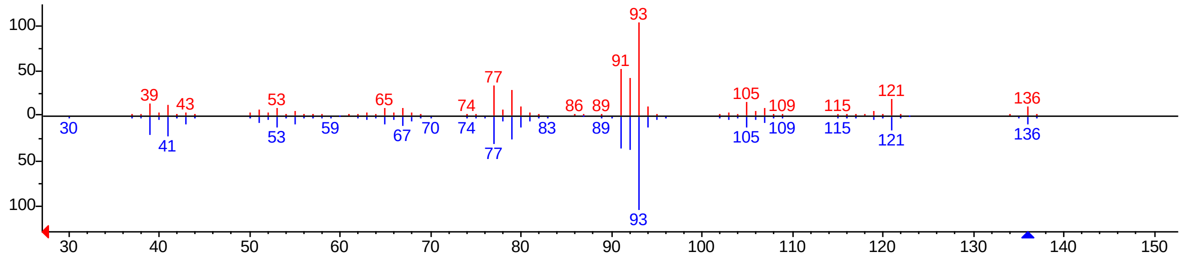
93
91
77
39 43 53 65 74 86 89 105 109 115 121 136
30 41 53 59 67 70 74 77 83 89 105 109 115 121 136
93

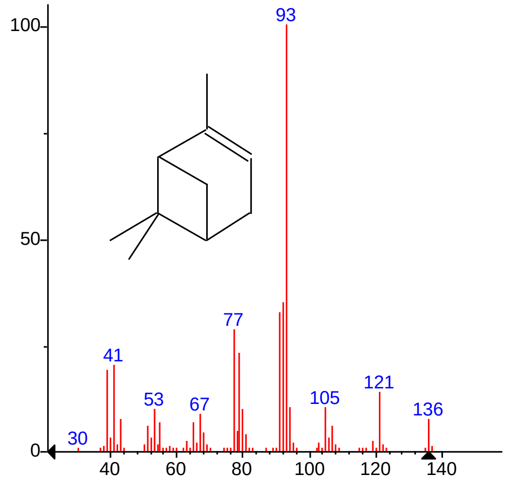
93
77
41
53 67 105 121 136
30

Camphene

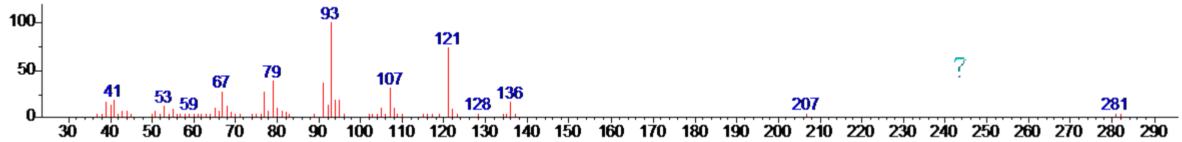
93
121
41 53 59 67 79 107 128 136 207 281
?

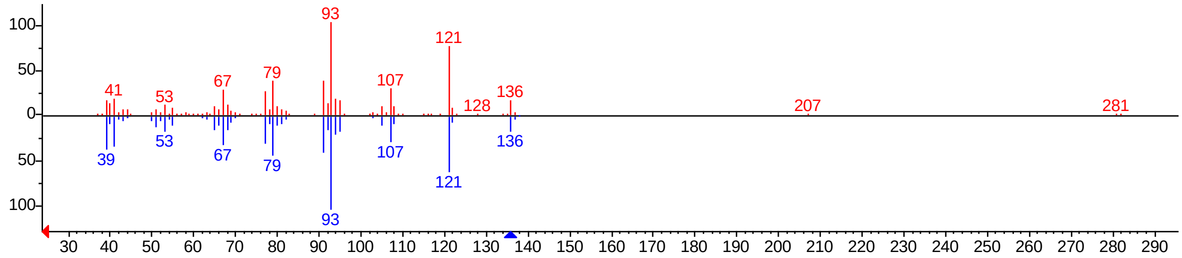
93
121
41 53 67 79 107 128 136 207 281
39 53 67 79 107 121 136
93

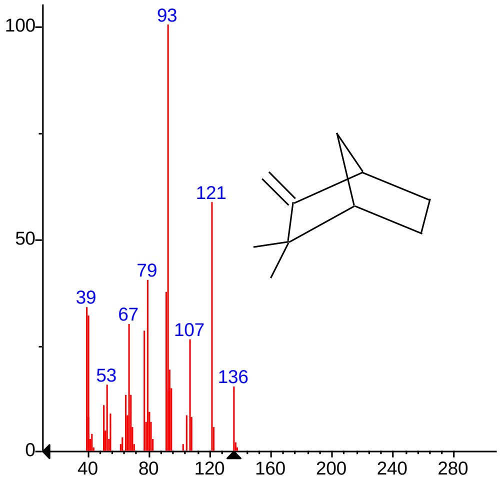

β-Phellandrene

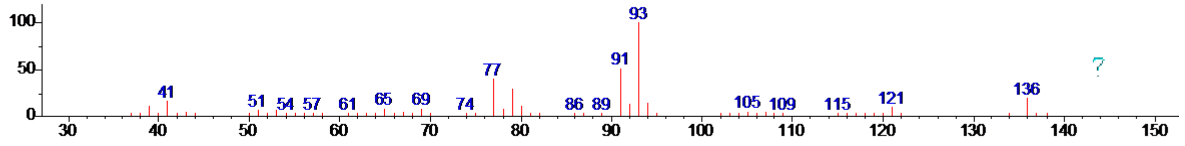

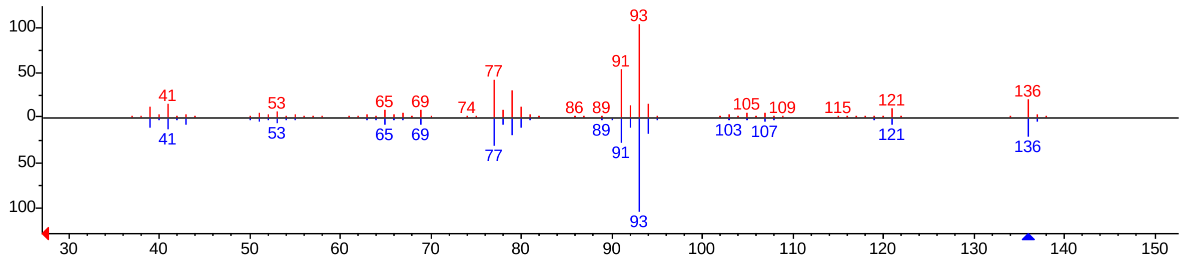

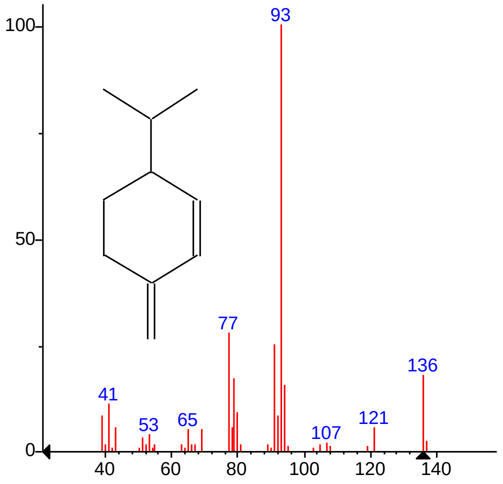

β –Thujene

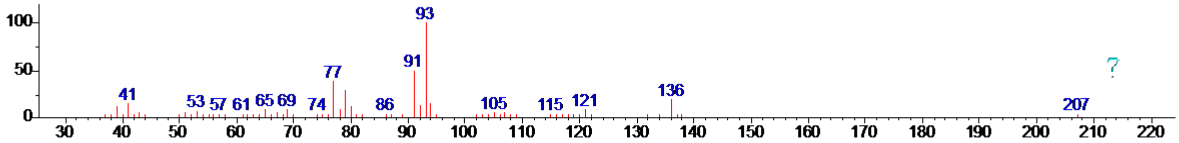

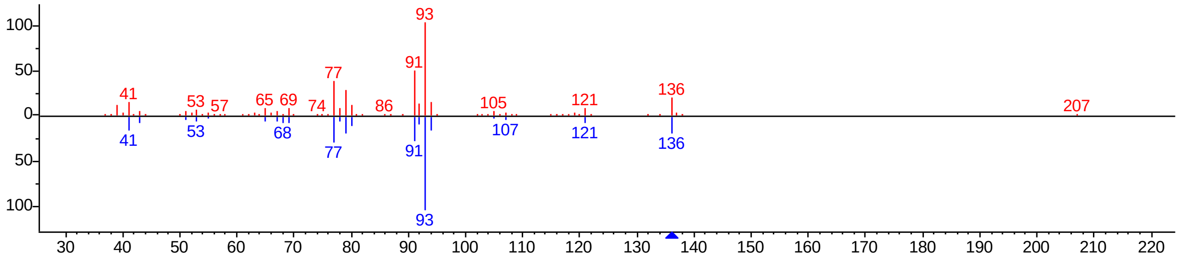

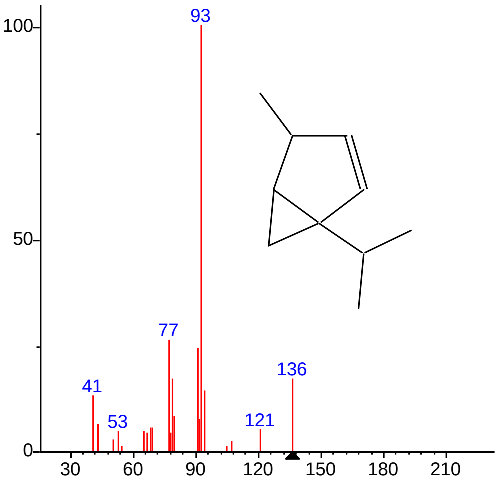

β-Pinene

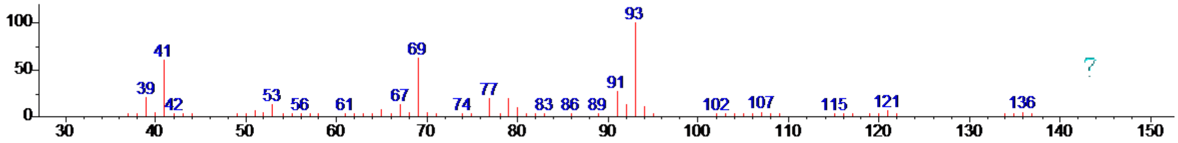

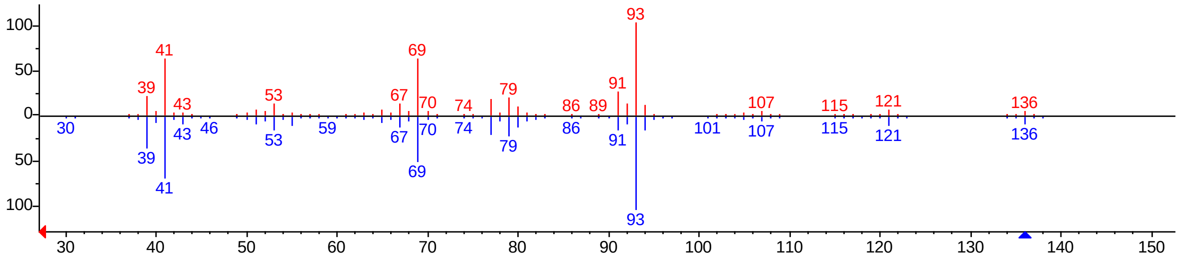

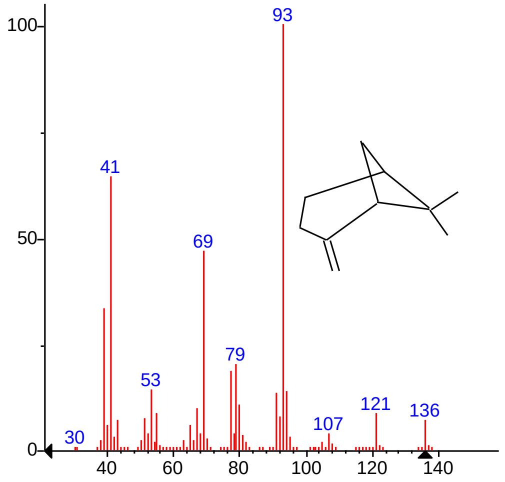

α-Phellandrene

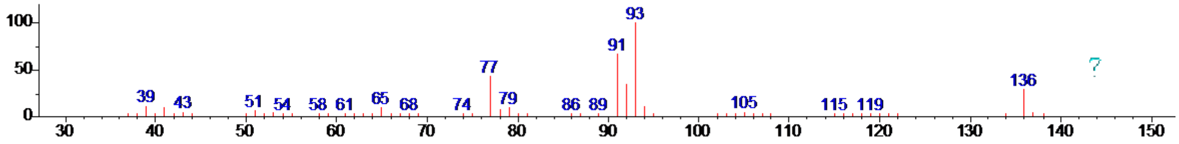

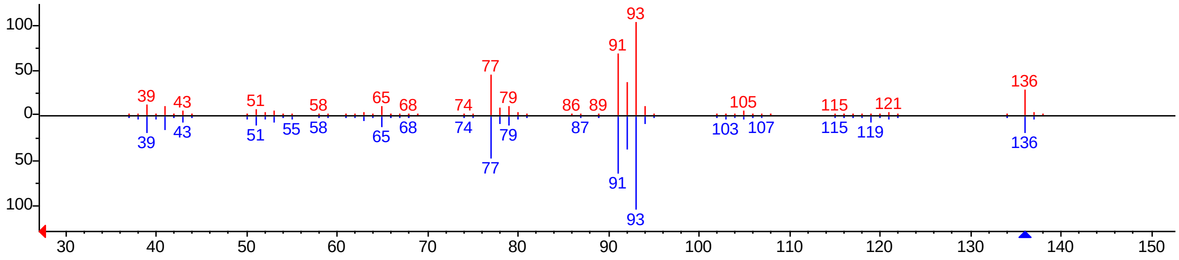

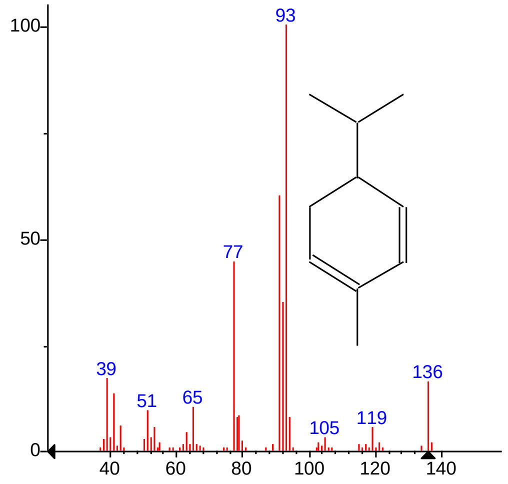
100
50
0
93
77
39
51
65
105
119
136
40
60
80
100
120
140

α-Terpinene

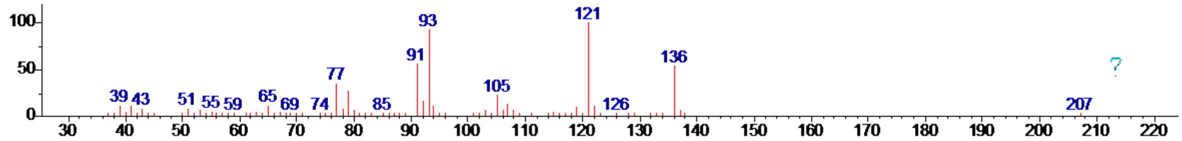
100
50
0
39 43 51 55 59 65 69 74 77 85 91 93 105 121 126 136 207
?
30 40 50 60 70 80 90 100 110 120 130 140 150 160 170 180 190 200 210 220

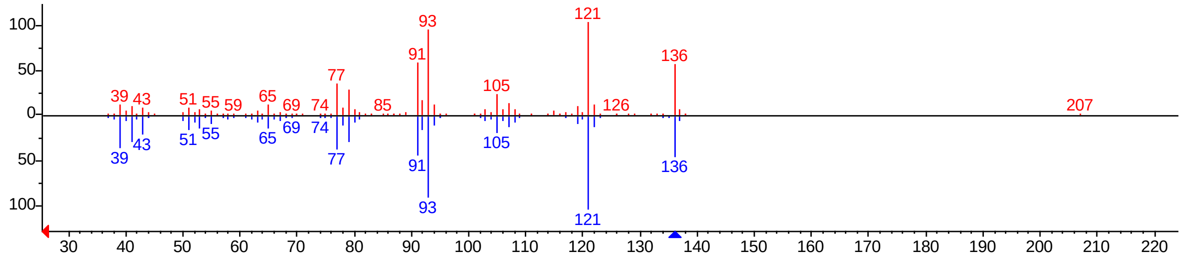
100
50
0
50
100
39 43 51 55 59 65 69 74 77 85 91 93 105 121 126 136 207
39 43 51 55 65 69 74 77 91 93 105 121 136
30 40 50 60 70 80 90 100 110 120 130 140 150 160 170 180 190 200 210 220

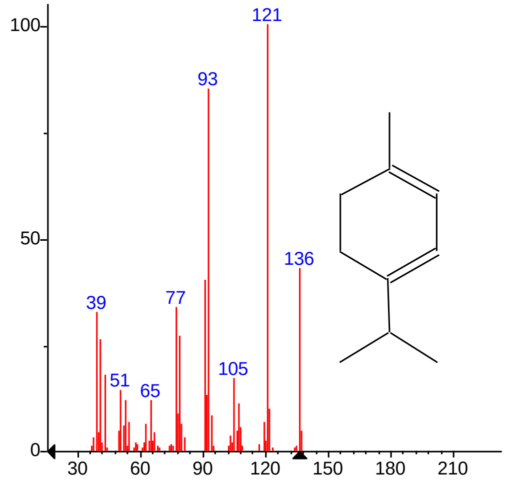
100
50
0
121
93
136
39
77
51
65
105
30
60
90
120
150
180
210

p-Cymene

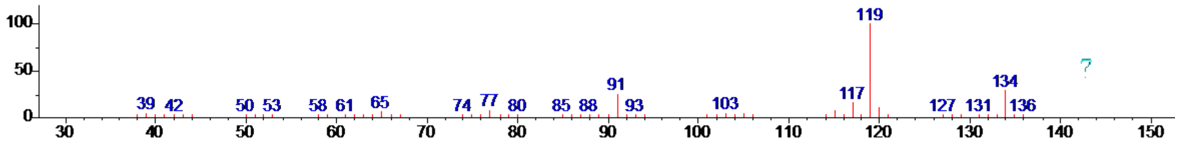

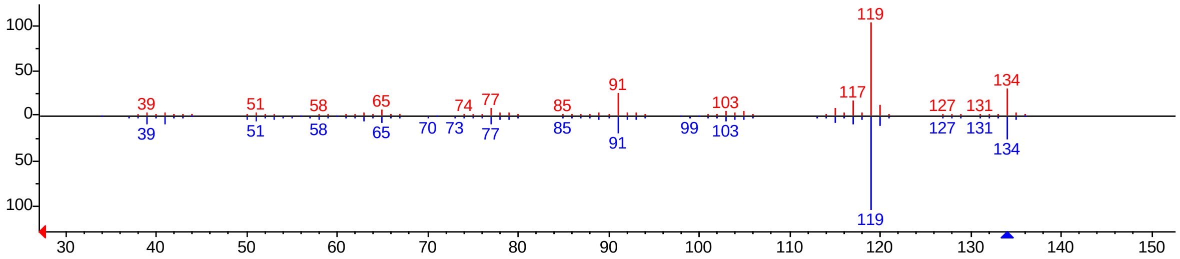

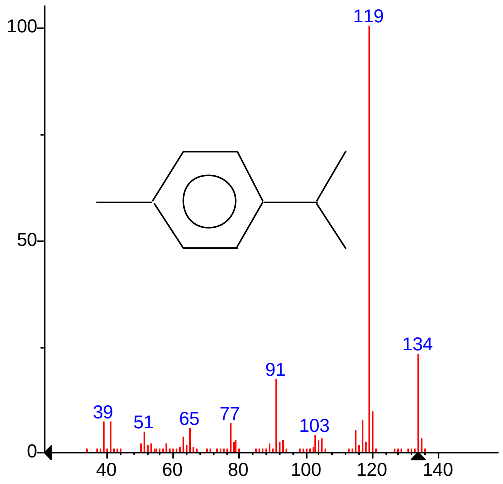

Υ-Terpinene

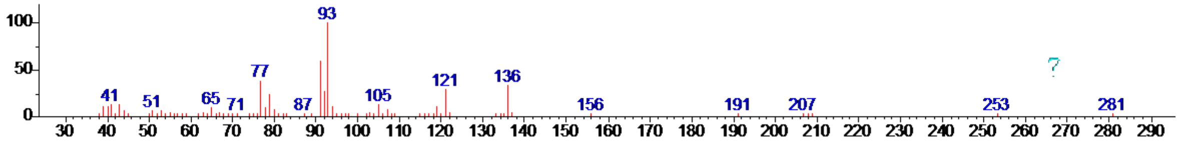

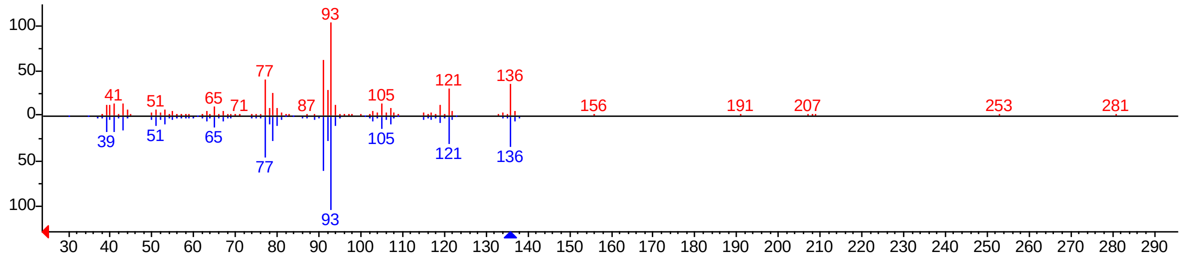

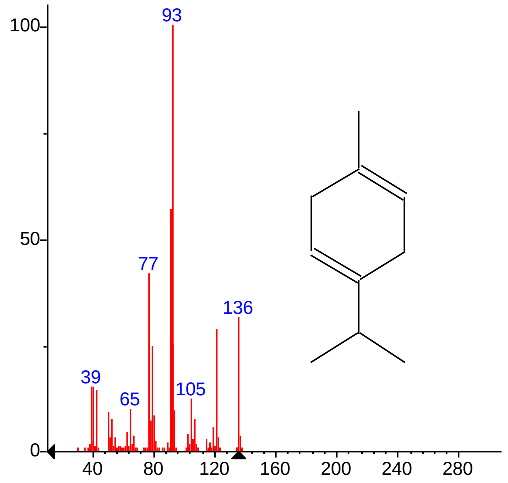

(±)-α-Terpinyl acetate

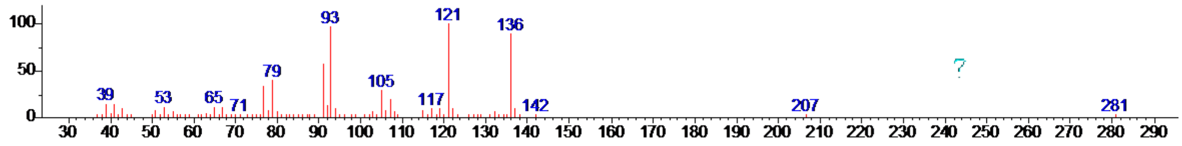

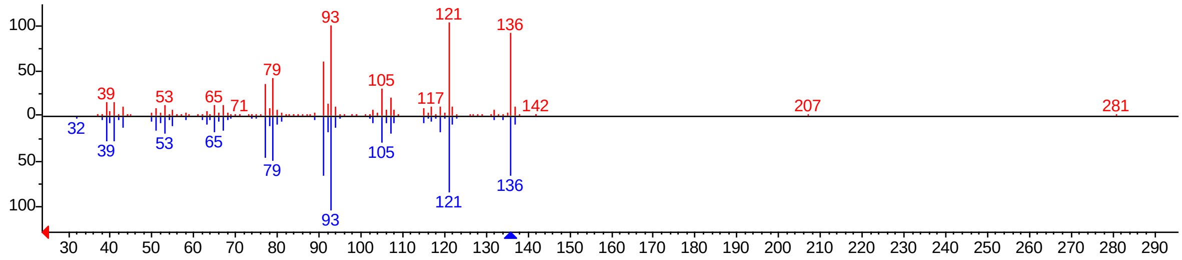

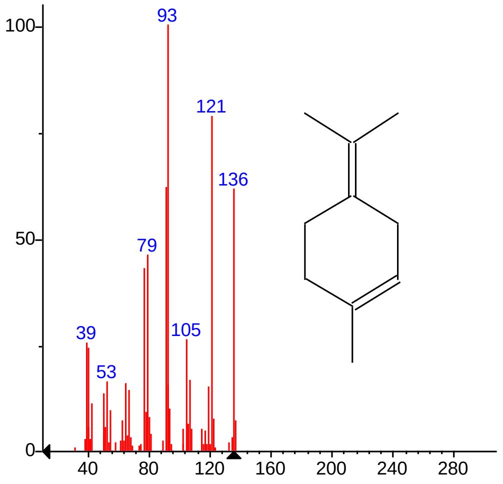

(+)-trans-4-Thujanol

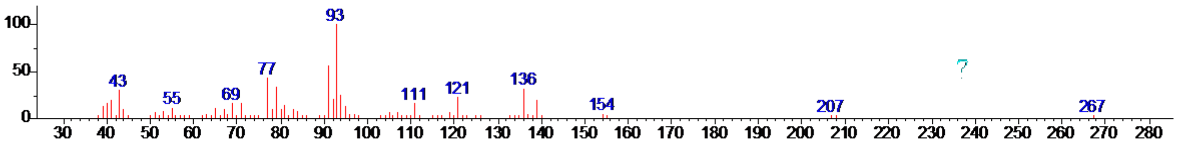

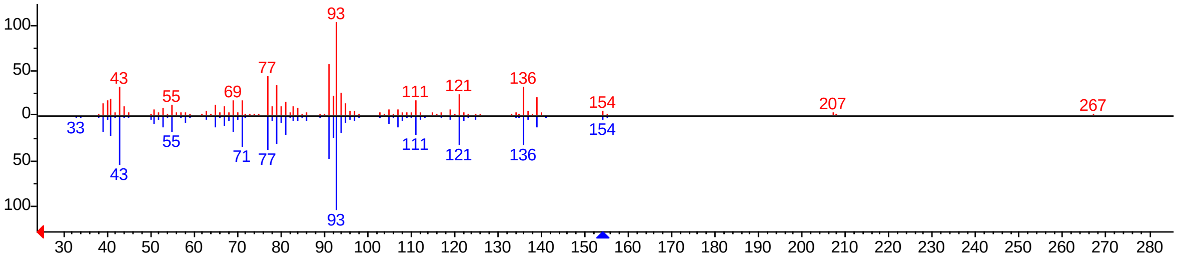

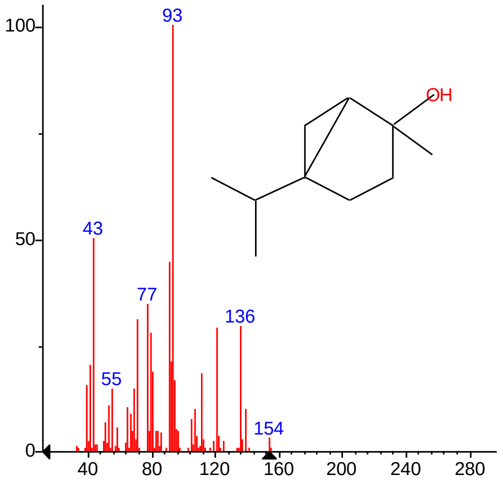

(±)-4-Terpineol

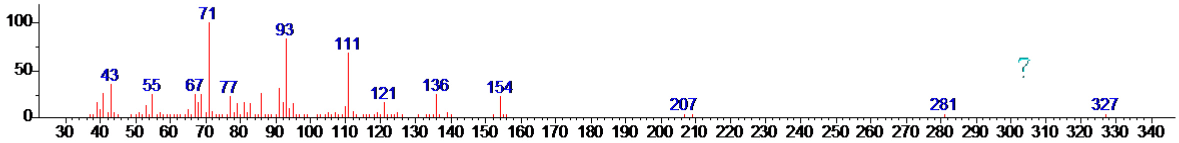

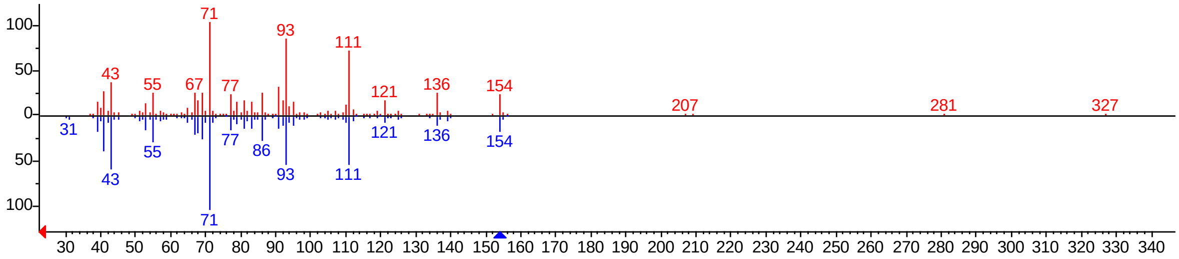

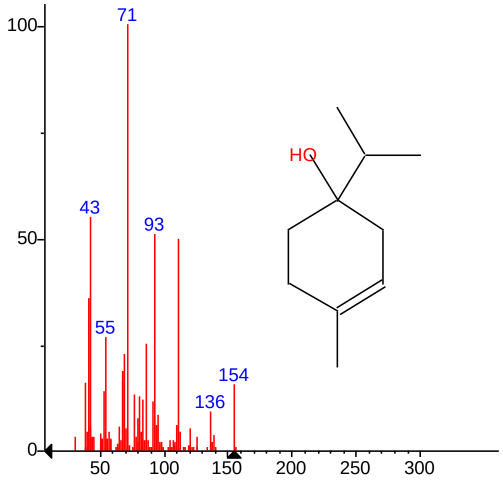

β-Caryophyllene

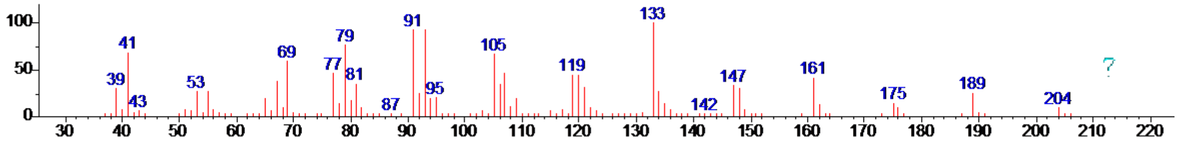

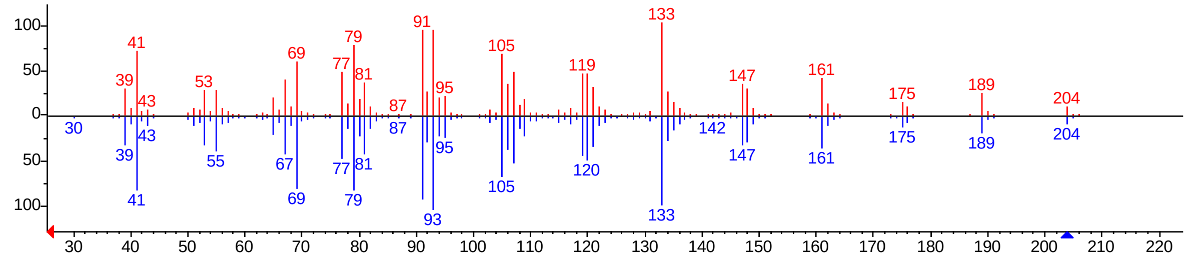

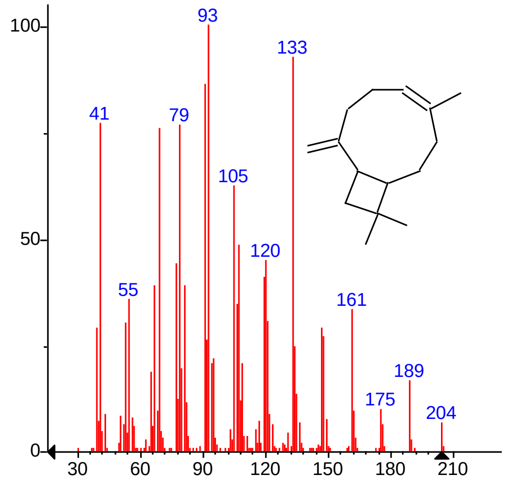

α-Caryophyllene

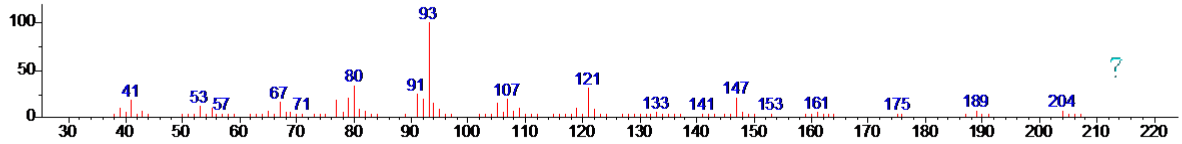

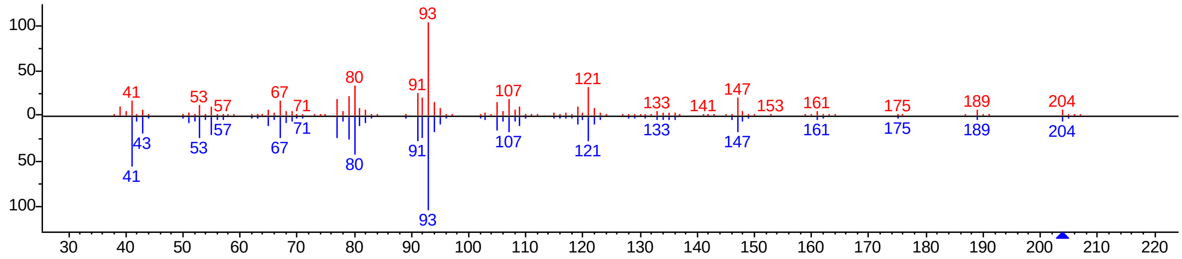

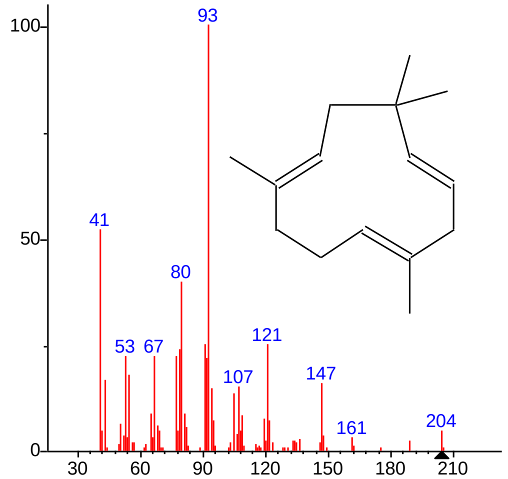

Germacrene D

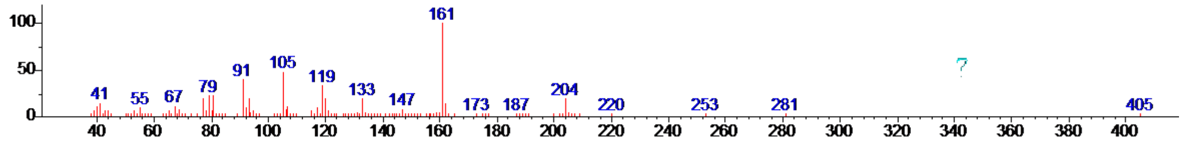

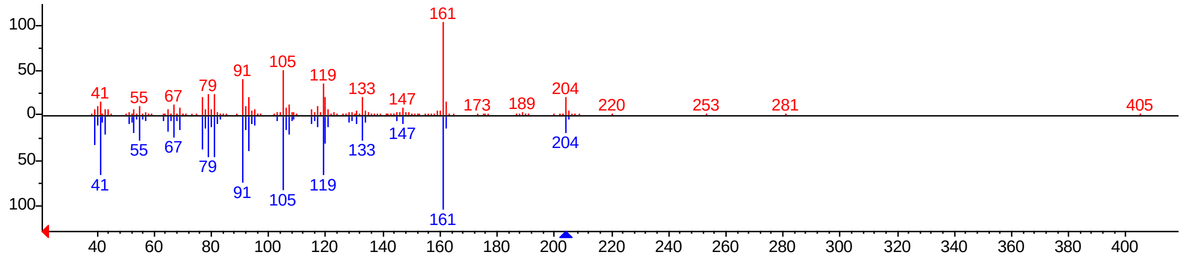

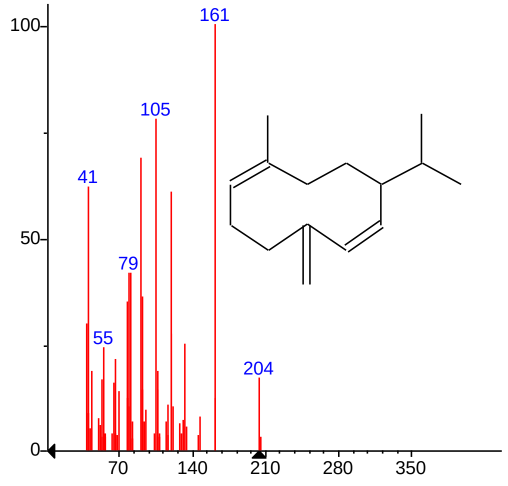

(-)-β-Cubebene

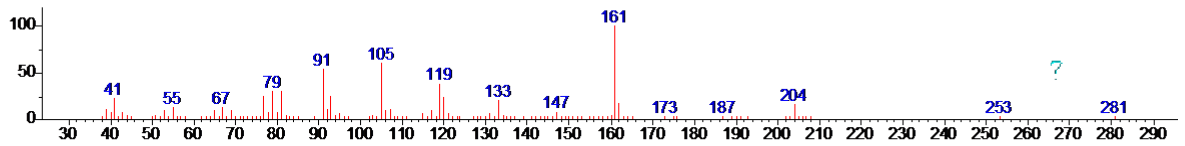
100
50
0
41
55
67
79
91
105
119
133
147
161
173
187
204
253
?
281
30 40 50 60 70 80 90 100 110 120 130 140 150 160 170 180 190 200 210 220 230 240 250 260 270 280 290

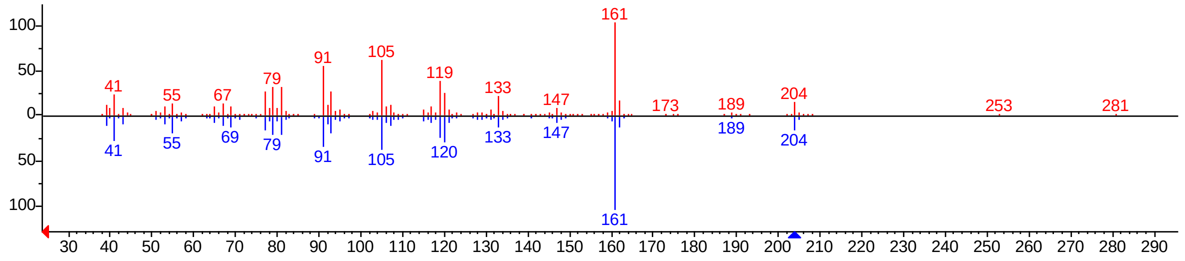
100
50
0
50
100
41
55
67
79
91
105
119
133
147
161
173
189
204
253
281
41
55
69
79
91
105
120
133
147
161
189
204
30 40 50 60 70 80 90 100 110 120 130 140 150 160 170 180 190 200 210 220 230 240 250 260 270 280 290

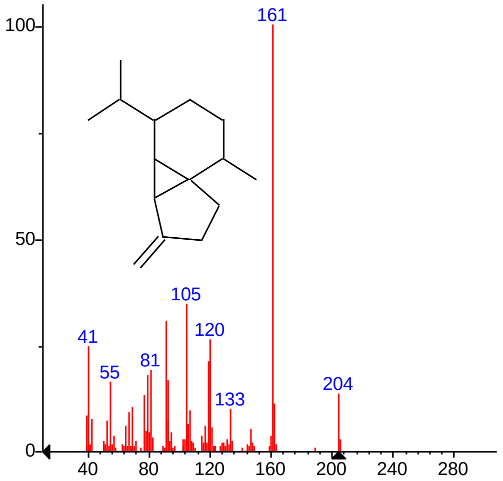
100
50
0
161
105
41
55
81
120
133
204
40 80 120 160 200 240 280

Lippia graveolens essential oil

Ethyl 2-methylbutanoate

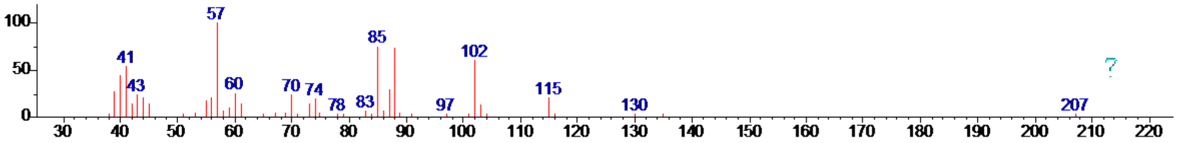

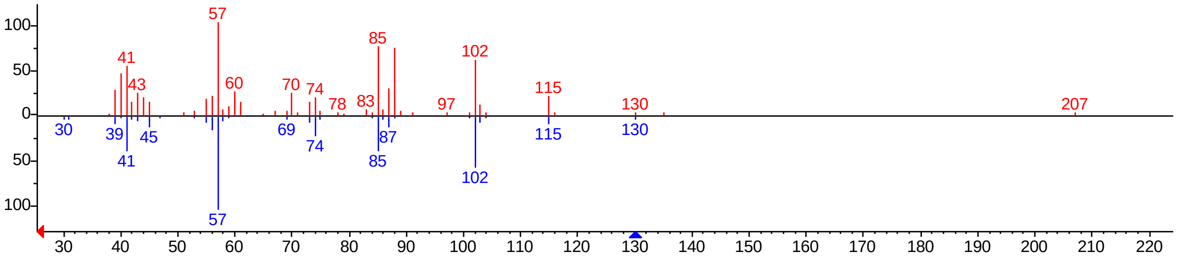

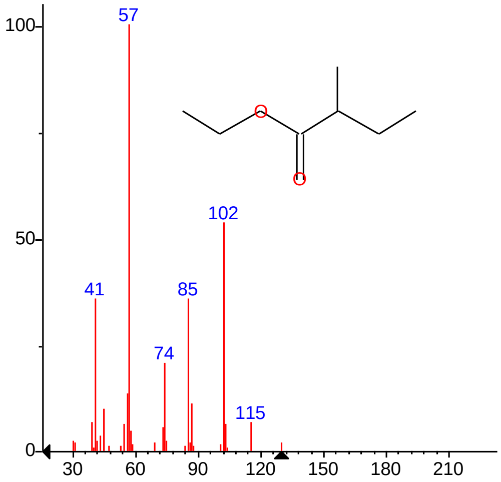

Sabinene

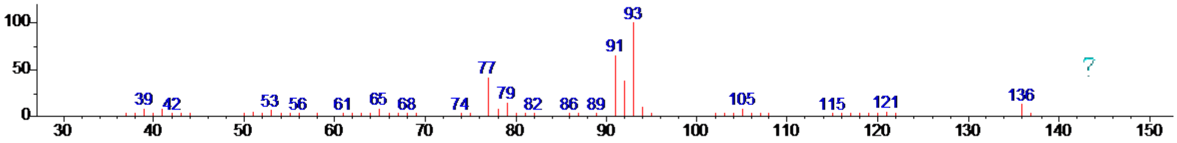

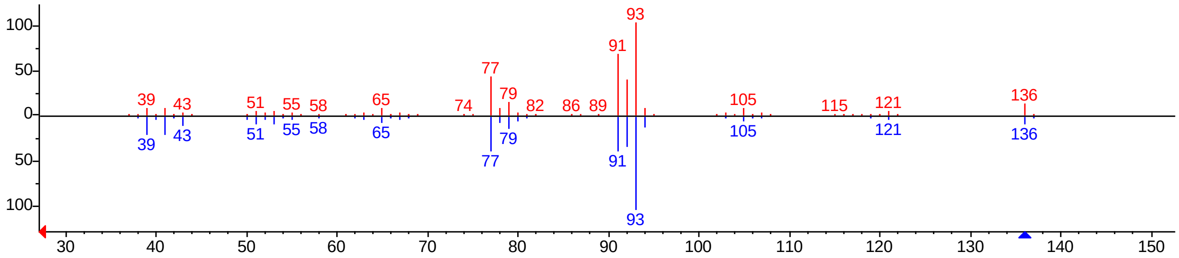
93
91
77
79
39 43 51 55 58 65 74 82 86 89 105 115 121 136
39 43 51 55 58 65 77 79 91 93 105 121 136
100 50 0 50 100
30 40 50 60 70 80 90 100 110 120 130 140 150

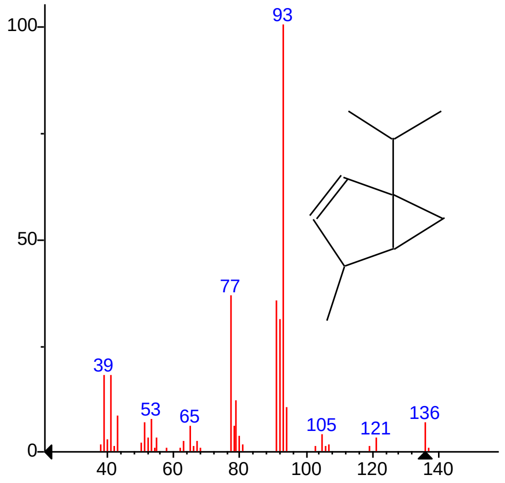
93
77
39 53 65 105 121 136
100 50 0
40 60 80 100 120 140

α-Thujene

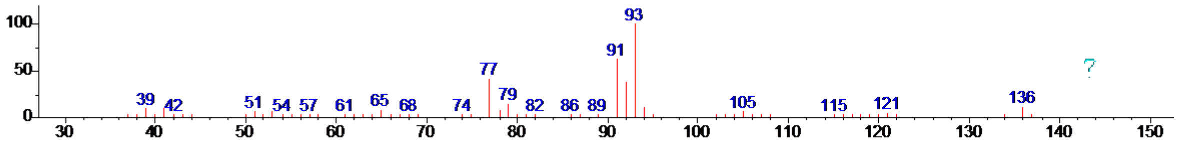
93
91
77
79
39 42 51 54 57 61 65 68 74 82 86 89 105 115 121 136
100 50 0
30 40 50 60 70 80 90 100 110 120 130 140 150

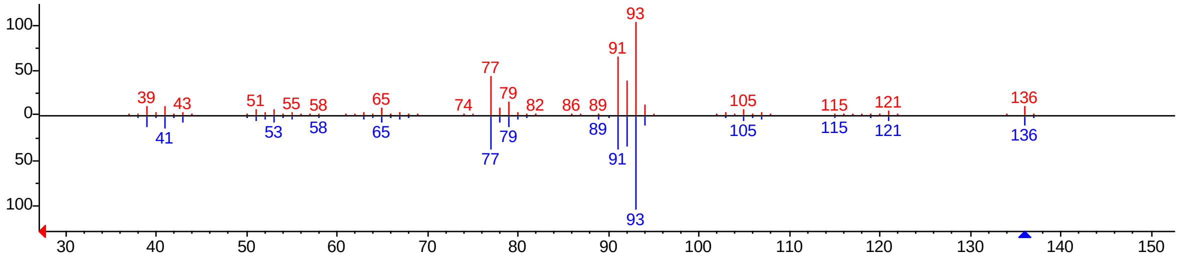
93
91
77
79
39 43 51 55 58 65 74 82 86 89 105 115 121 136
41 53 58 65 77 79 89 91 93 105 115 121 136
100 50 0 50 100
30 40 50 60 70 80 90 100 110 120 130 140 150

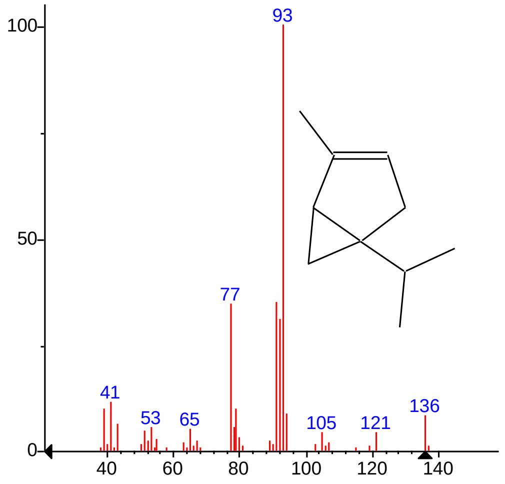
93
77
41
53
65
105
121
136
100
50
0
40
60
80
100
120
140

(±)-α-Pinene

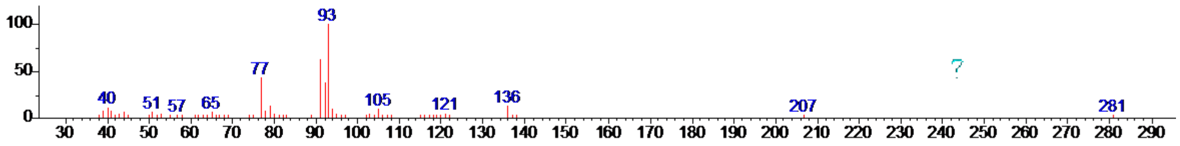
93
77
40
51
57
65
105
121
136
207
281
100
50
0
30 40 50 60 70 80 90 100 110 120 130 140 150 160 170 180 190 200 210 220 230 240 250 260 270 280 290

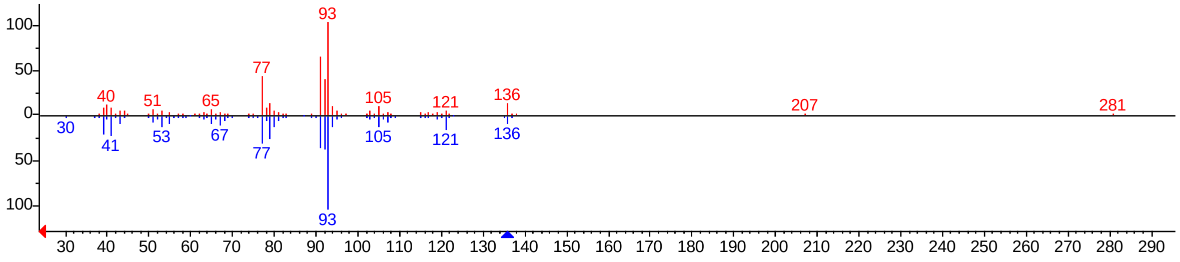
93
77
40
51
65
105
121
136
207
281
30
41
53
67
77
105
121
136
93
100
50
0
50
100
30 40 50 60 70 80 90 100 110 120 130 140 150 160 170 180 190 200 210 220 230 240 250 260 270 280 290

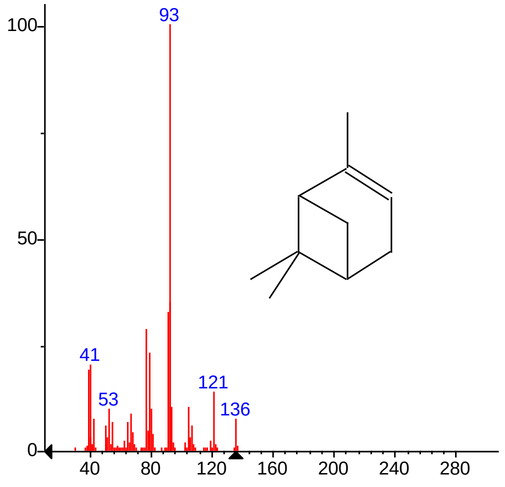
93
41
53
121
136
100
50
0
40
80
120
160
200
240
280

β-Thujene

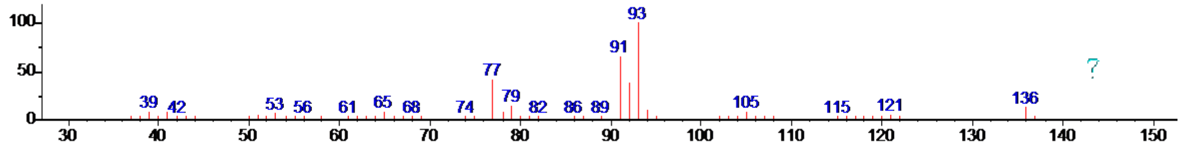

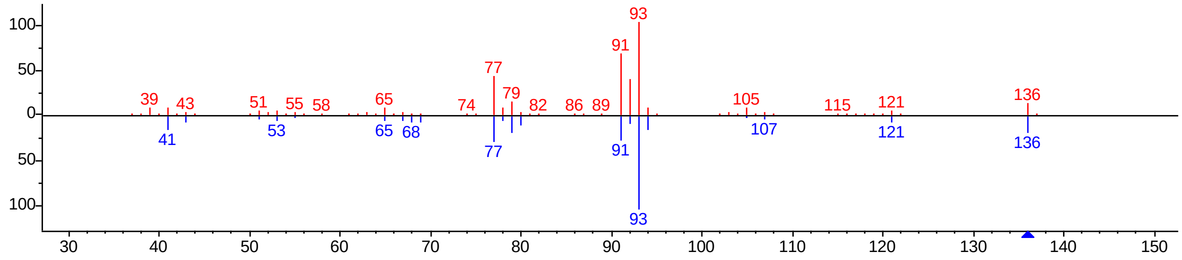

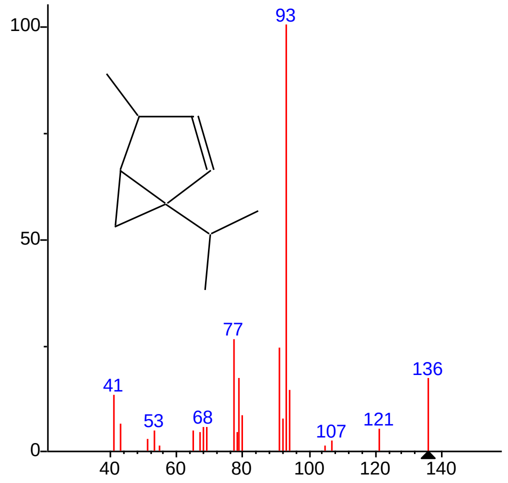

Camphene

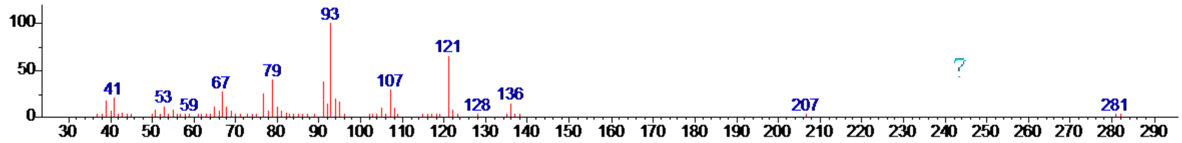

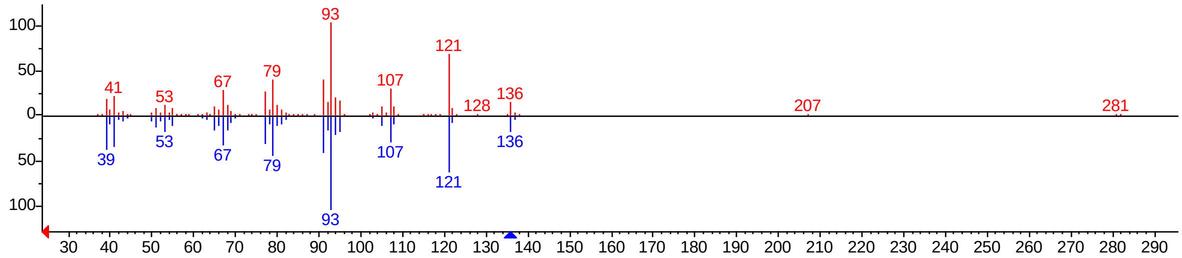

100
50
0
50
100
93
121
41
53
67
79
107
128
136
207
281
39
53
67
79
93
107
121
136
30
40
50
60
70
80
90
100
110
120
130
140
150
160
170
180
190
200
210
220
230
240
250
260
270
280
290

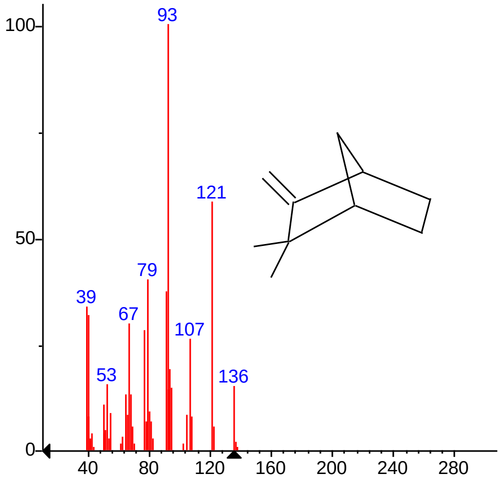

100
50
0
93
121
39
53
67
79
107
136
40
80
120
160
200
240
280

(±)-β-Pinene

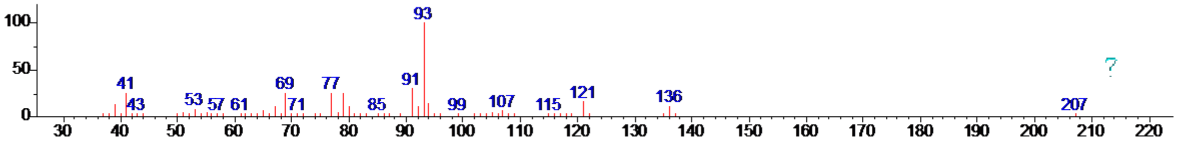

100
50
0
93
41
43
53
57
61
69
71
77
85
91
99
107
115
121
136
207
30
40
50
60
70
80
90
100
110
120
130
140
150
160
170
180
190
200
210
220

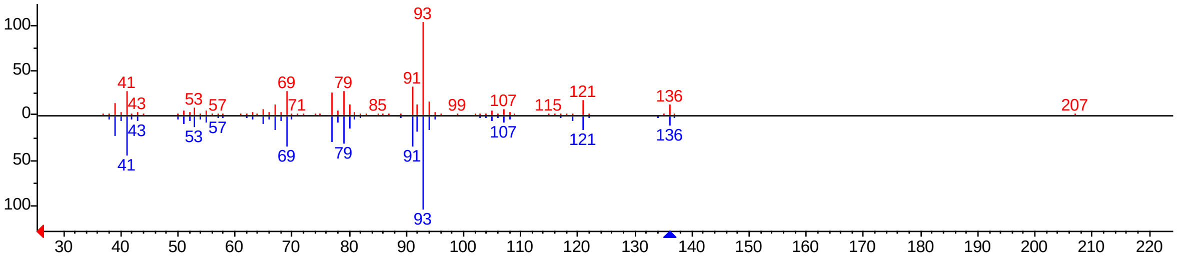

100
50
0
50
100
93
41
43
53
57
69
71
79
85
91
99
107
115
121
136
207
43
41
53
57
69
79
91
93
107
121
136
30
40
50
60
70
80
90
100
110
120
130
140
150
160
170
180
190
200
210
220

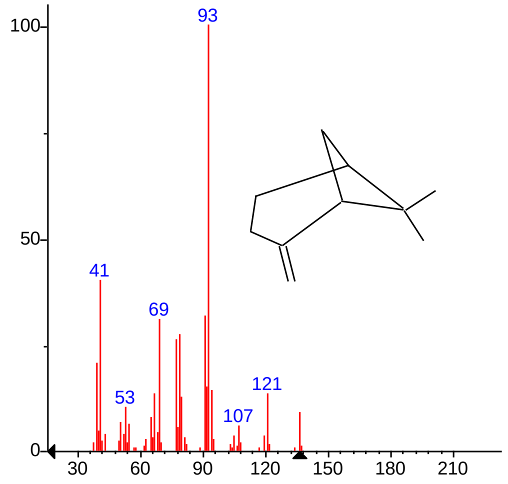
93
41
69
53
121
107
100
50
0
30 60 90 120 150 180 210

β-Pinene

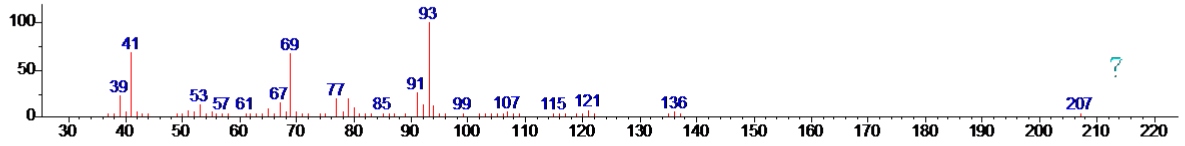
93
41
69
39
53 57 61
67
77
91
85
99
107
115
121
136
207
?
100
50
0
30 40 50 60 70 80 90 100 110 120 130 140 150 160 170 180 190 200 210 220

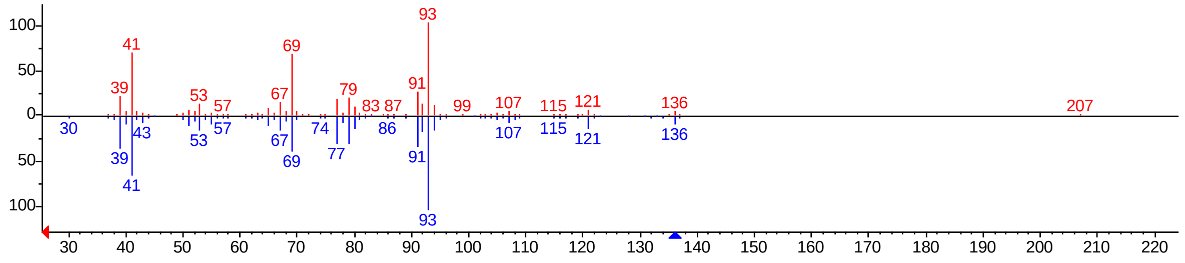
93
41
69
39
53 57
67
79
83 87
91
99
107
115
121
136
207
30
43
39
41
53
57
67
69
74
77
86
91
93
107
115
121
136
100
50
0
50
100
30 40 50 60 70 80 90 100 110 120 130 140 150 160 170 180 190 200 210 220

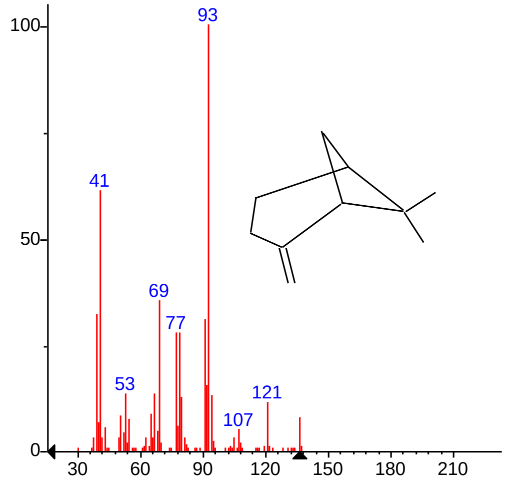
93
41
69
77
53
121
107
100
50
0
30 60 90 120 150 180 210

α-Phellandrene

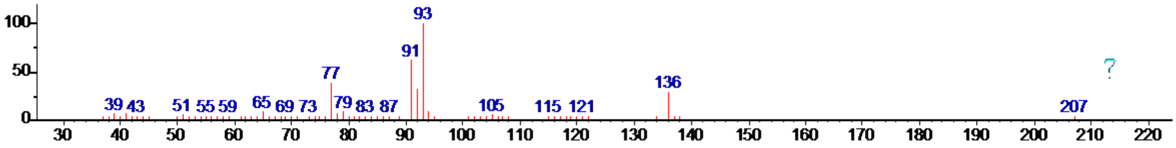

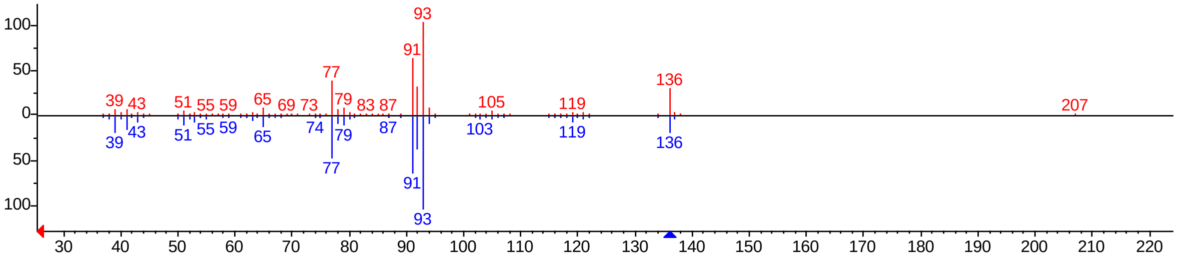

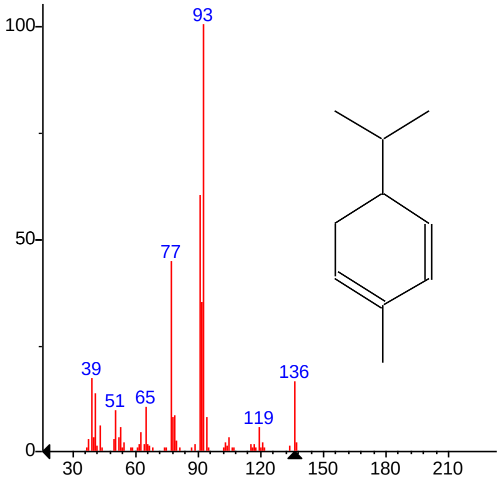

(±)-3-Carene

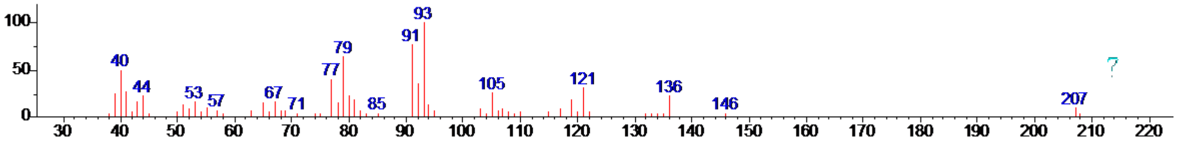

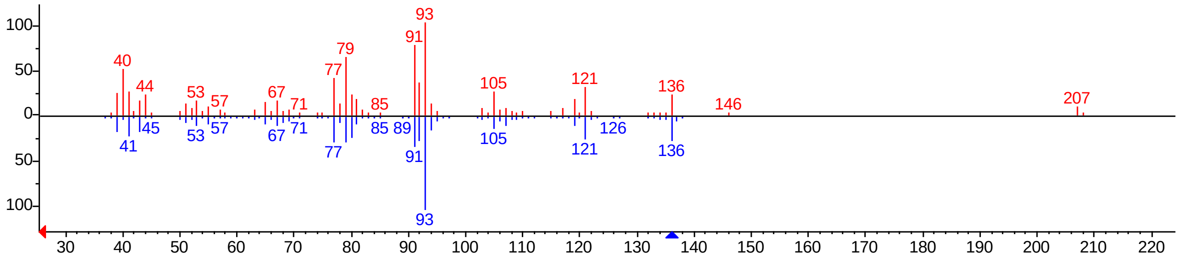

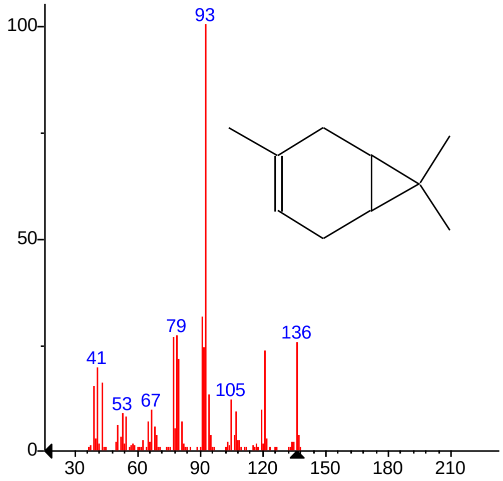

(±)-α-Terpinyl acetate

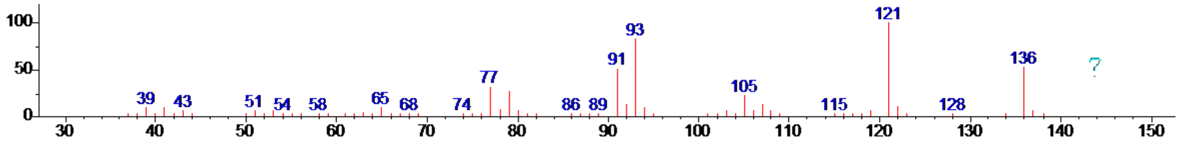

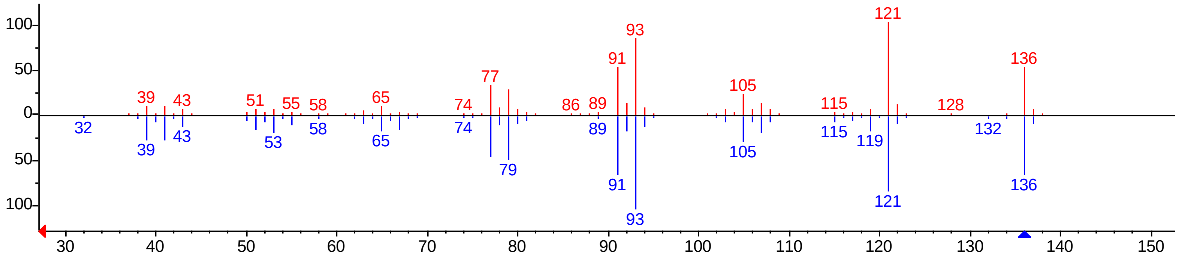

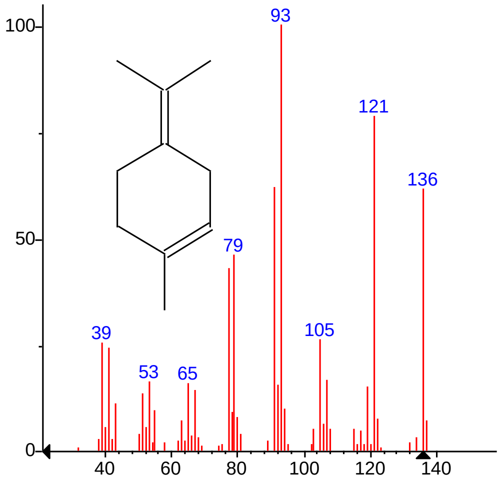
100
50
0
93
121
136
79
39
53
65
105
40
60
80
100
120
140

p-Cymene

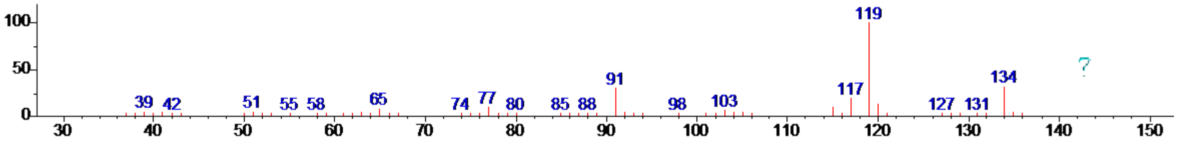
100
50
0
119
91
134
117
39
42
51
55
58
65
74
77
80
85
88
98
103
127
131
30
40
50
60
70
80
90
100
110
120
130
140
150

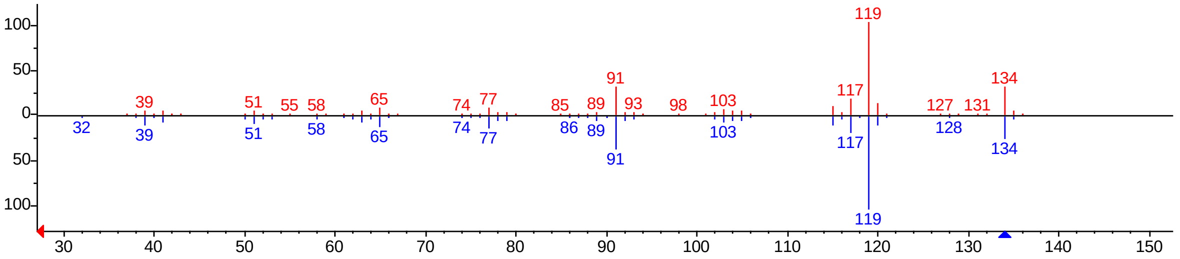
100
50
0
50
100
119
91
134
117
39
51
55
58
65
74
77
85
89
93
98
103
127
131
32
39
51
58
65
74
77
86
89
91
103
117
128
134
119
30
40
50
60
70
80
90
100
110
120
130
140
150

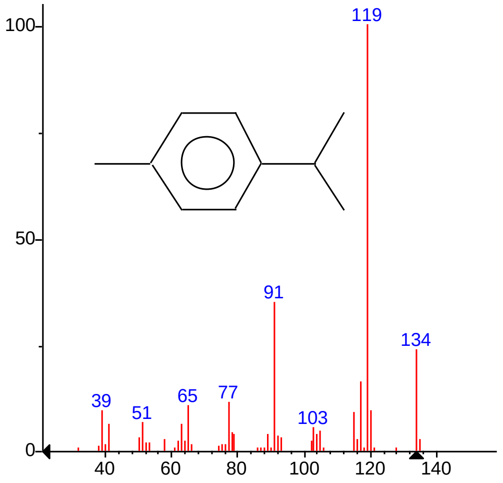
100
50
0
119
91
134
39
51
65
77
103
40
60
80
100
120
140

(±)-Eucalyptol

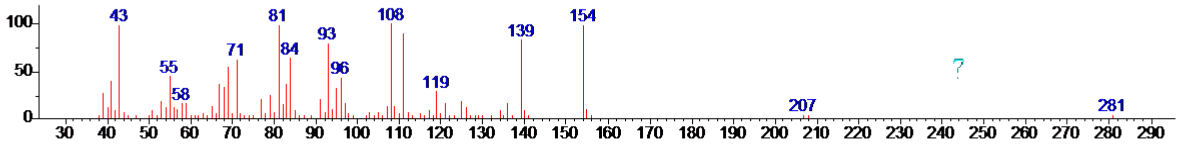

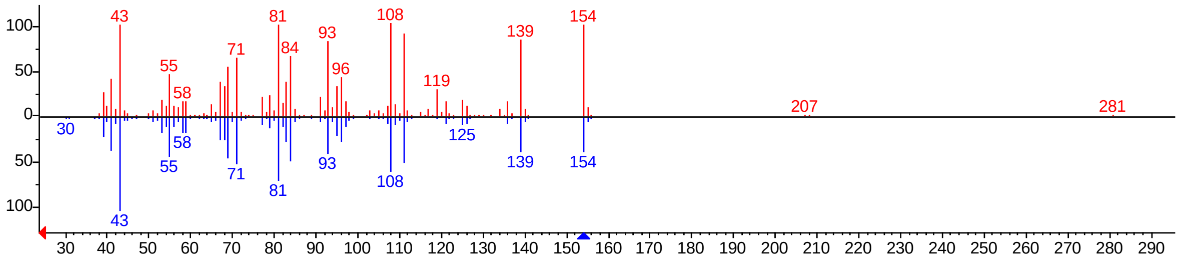

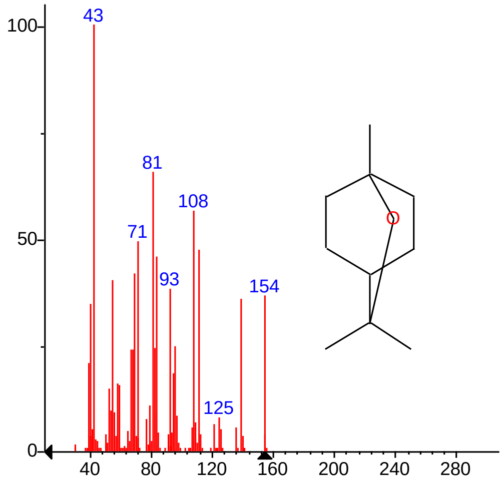

Ocimene

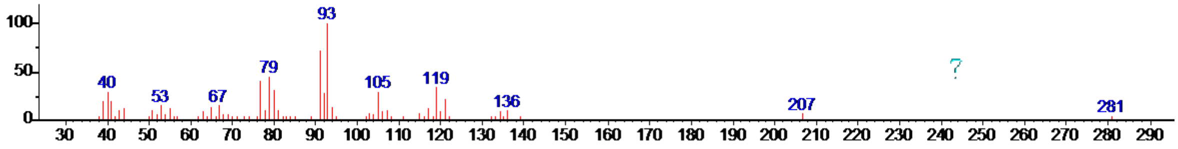

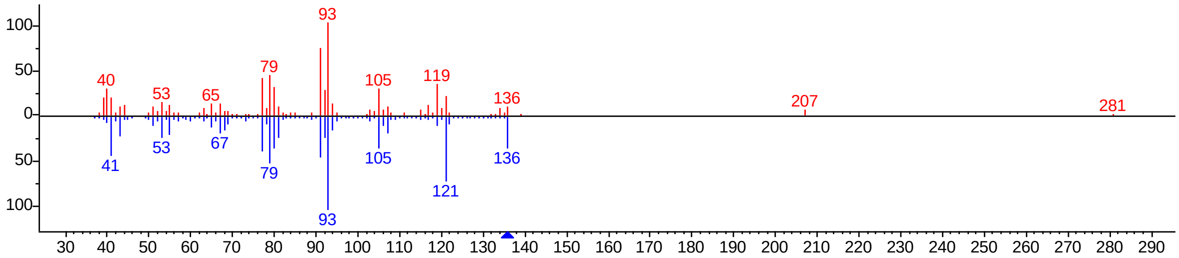

100
50
0
50
100
93
40
53
65
79
105
119
136
207
281
41
53
67
79
105
121
136
93
30 40 50 60 70 80 90 100 110 120 130 140 150 160 170 180 190 200 210 220 230 240 250 260 270 280 290

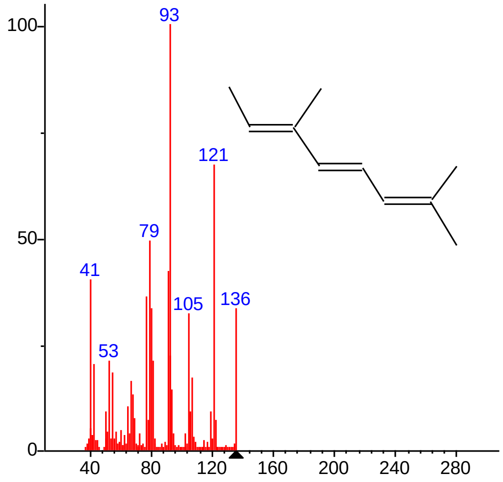

100
50
0
93
121
79
41
105
136
53
40 80 120 160 200 240 280

α-Terpinene

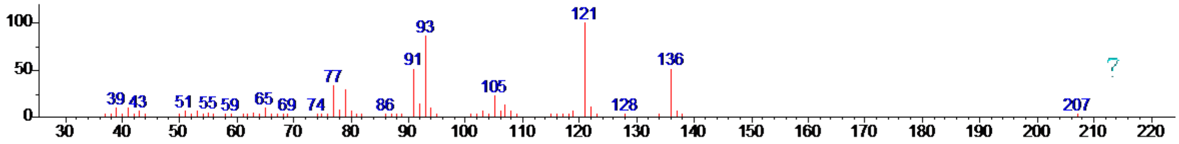

100
50
0
121
93
91
136
77
105
39 43 51 55 59 65 69 74 86 128 207
?
30 40 50 60 70 80 90 100 110 120 130 140 150 160 170 180 190 200 210 220

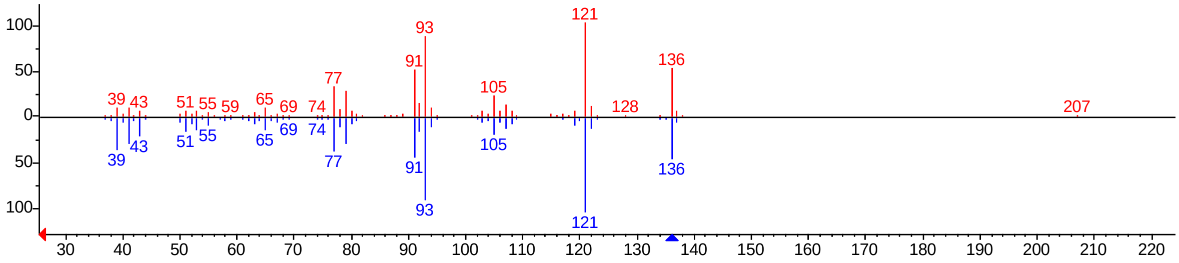

100
50
0
50
100
121
93
91
136
77
105
39 43 51 55 59 65 69 74 128 207
39 43 51 55 65 69 74 77 91 93 105 121 136
30 40 50 60 70 80 90 100 110 120 130 140 150 160 170 180 190 200 210 220

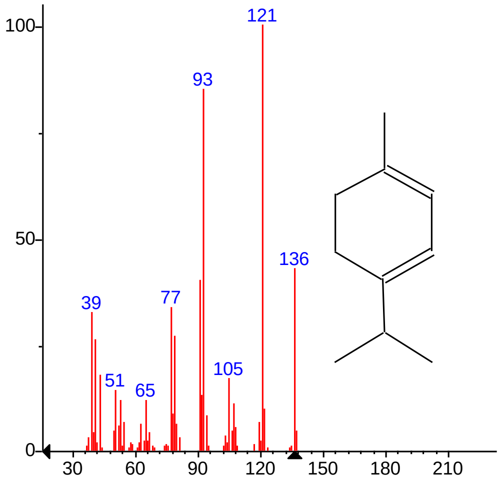

cis-(±)-4-Thujanol

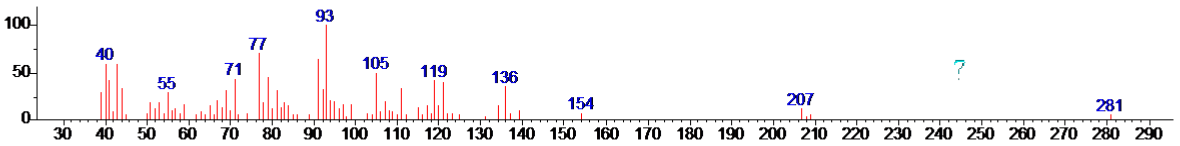

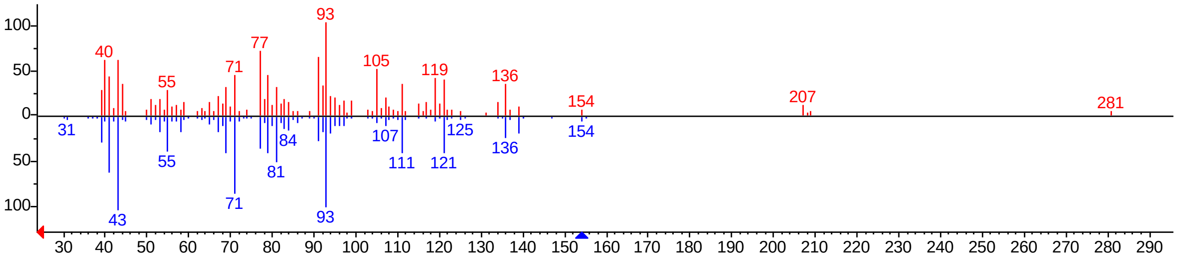

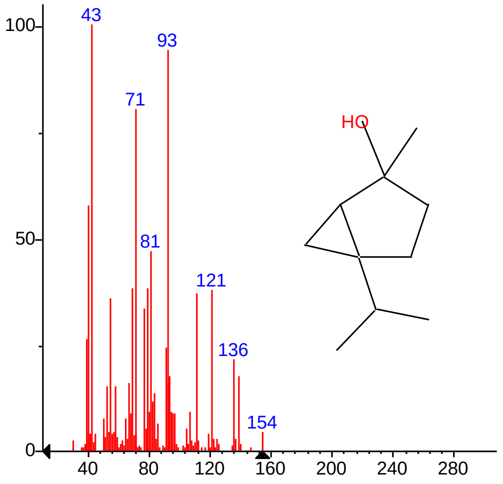

2-Carene

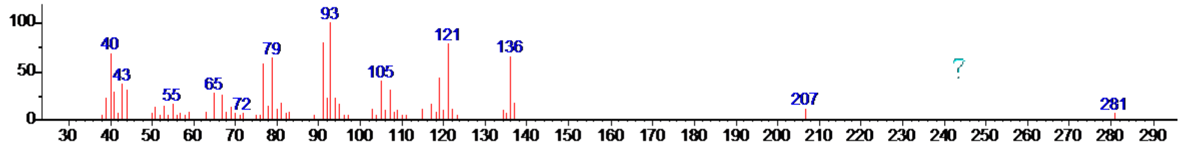

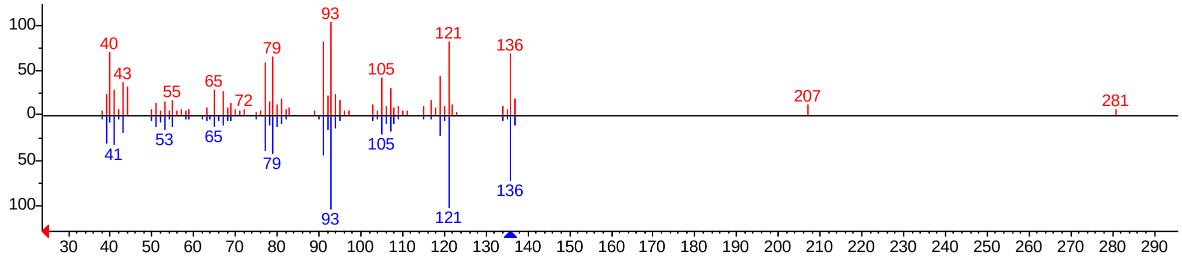

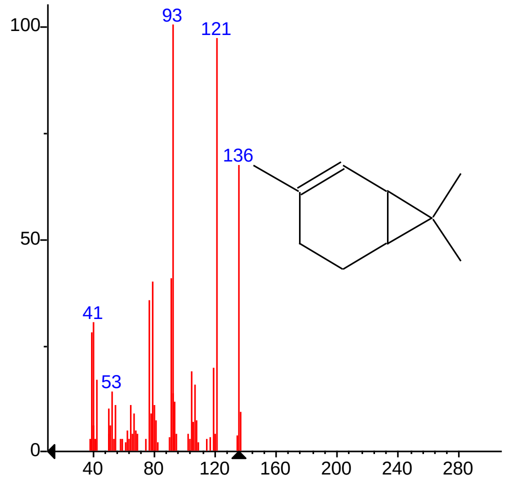

(±)-Linalool

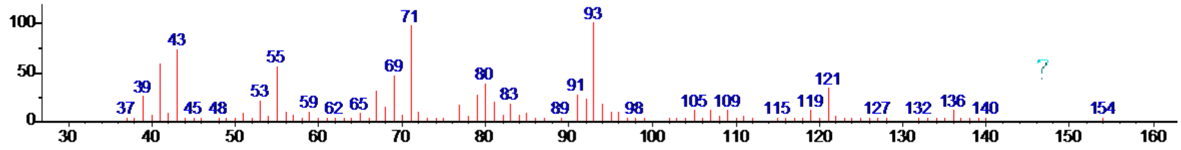

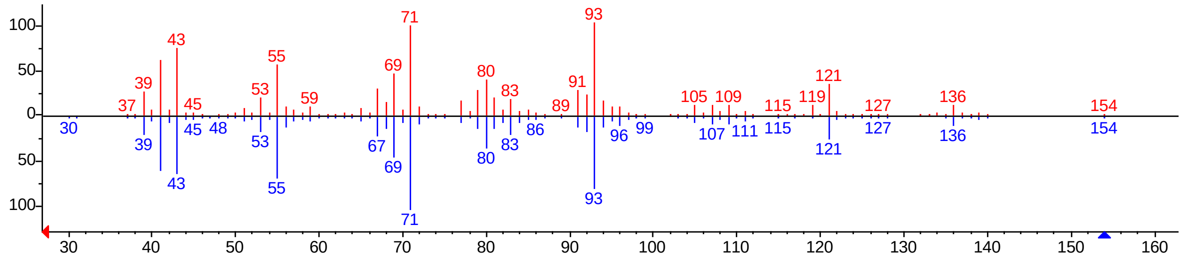

37 39 43 45 53 55 59 69 71 80 83 89 91 93 105 109 115 119 121 127 136 154
30 39 43 45 48 53 55 67 69 71 80 83 86 93 96 99 107 111 115 121 127 136 154

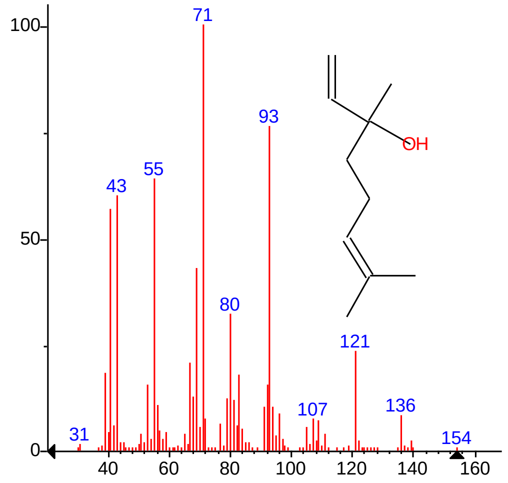

31 43 55 71 80 93 107 121 136 154
OH

(±)-Camphor

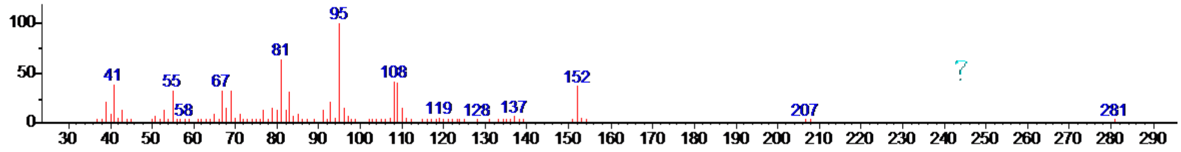

41 55 58 67 81 95 108 119 128 137 152 207 281

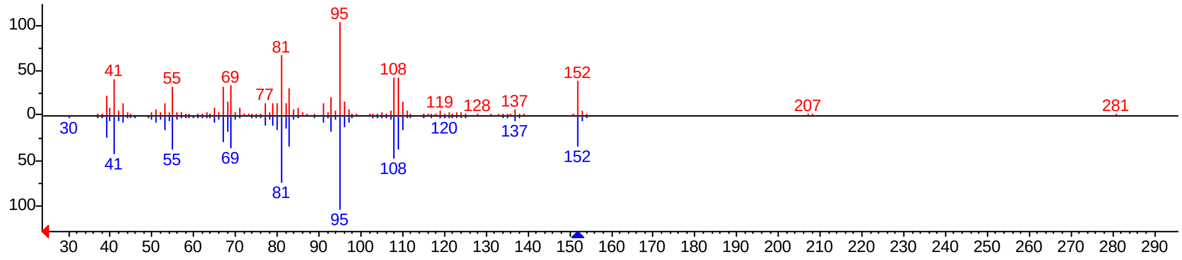

41 55 69 77 81 95 108 119 128 137 152 207 281
30 41 55 69 81 95 108 120 137 152

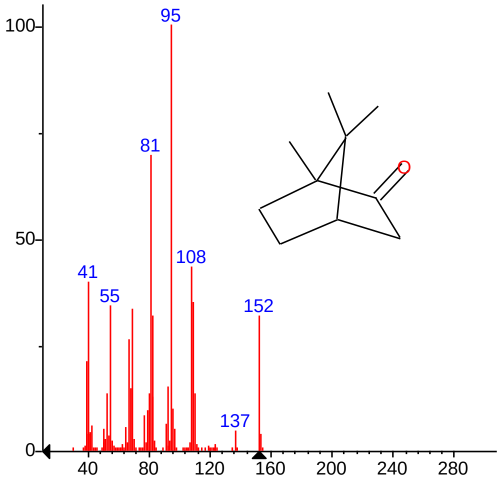
95
81
108
41
55
152
137
O
100
50
0
40
80
120
160
200
240
280

(+)-Borneol

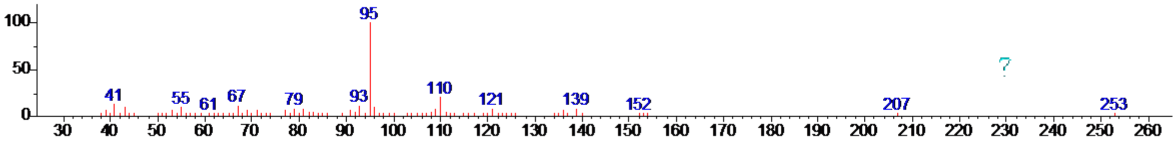
95
41
55
61
67
79
93
110
121
139
152
207
253
100
50
0
30 40 50 60 70 80 90 100 110 120 130 140 150 160 170 180 190 200 210 220 230 240 250 260

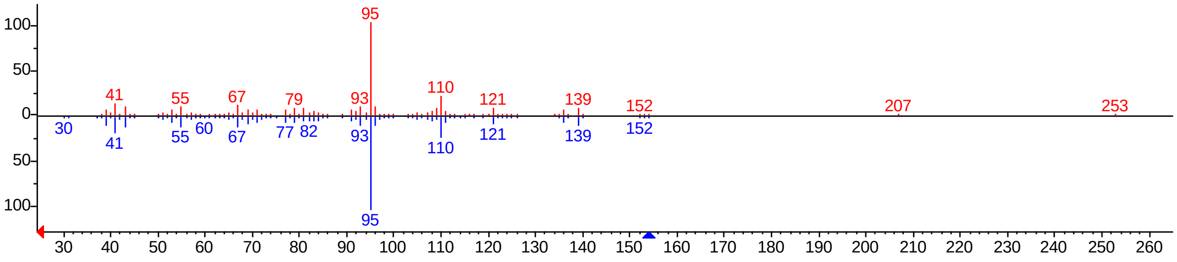
95
41
55
67
79
93
110
121
139
152
207
253
30
41
55
60
67
77
82
93
110
121
139
152
95
100
50
0
50
100
30 40 50 60 70 80 90 100 110 120 130 140 150 160 170 180 190 200 210 220 230 240 250 260

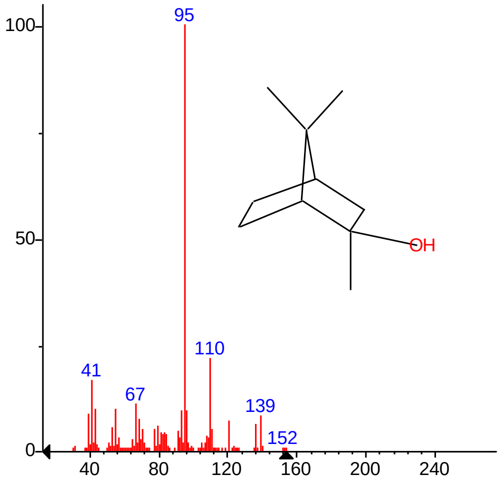
95
110
41
67
139
152
OH
100
50
0
40
80
120
160
200
240

(±)-4-Terpineol

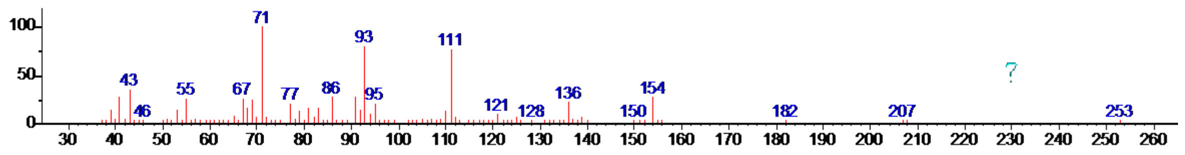

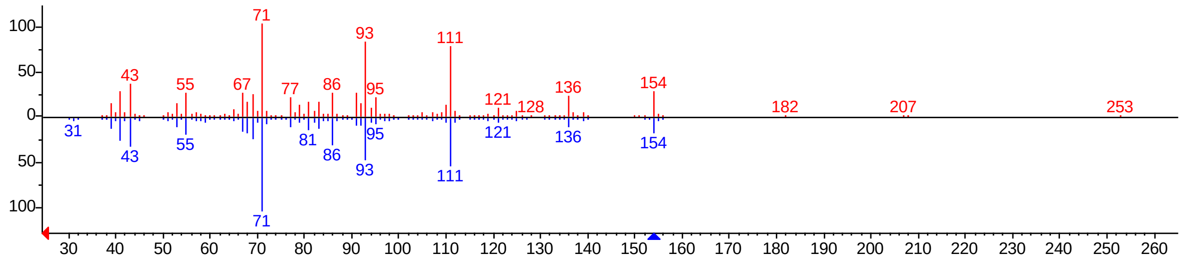

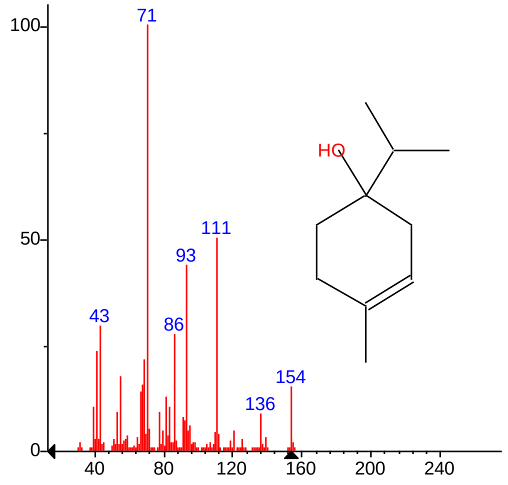

(±)-α-Terpineol

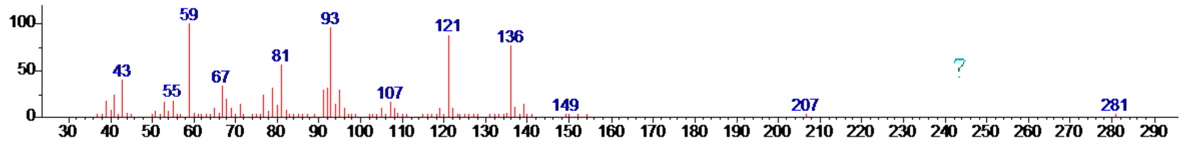

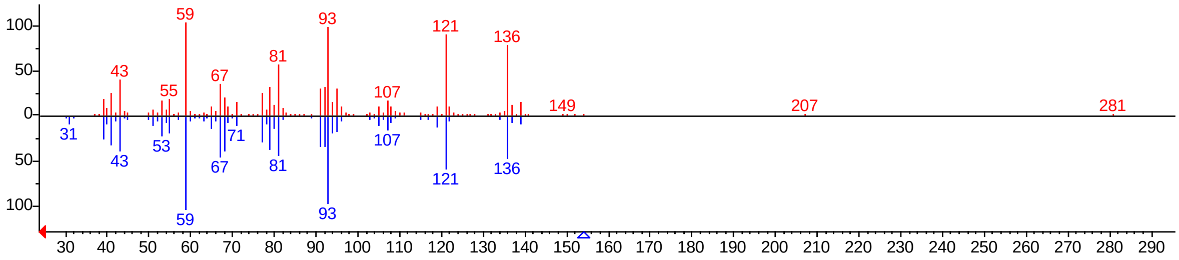

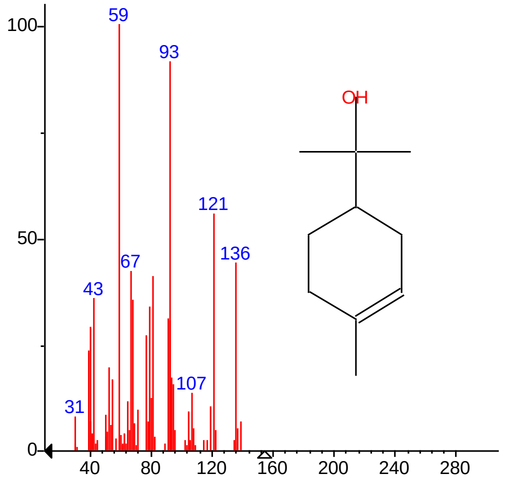

1-Isopropyl-2-methoxy-4-methylbenzene

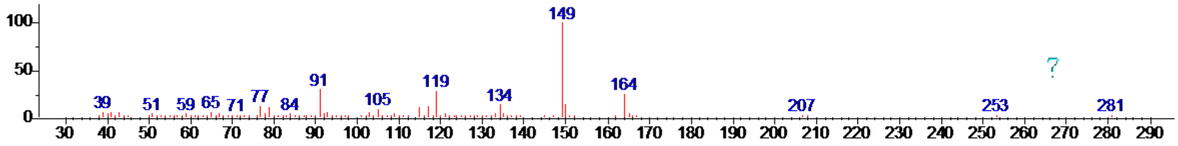

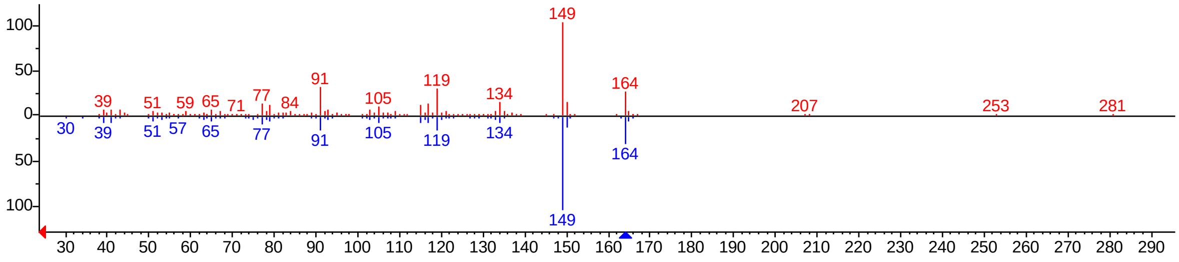

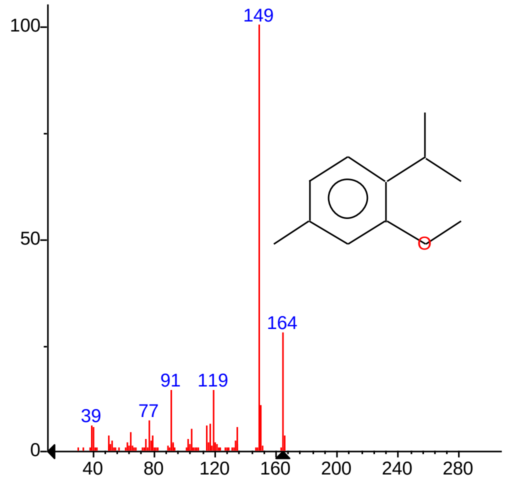

Carvotanacetone

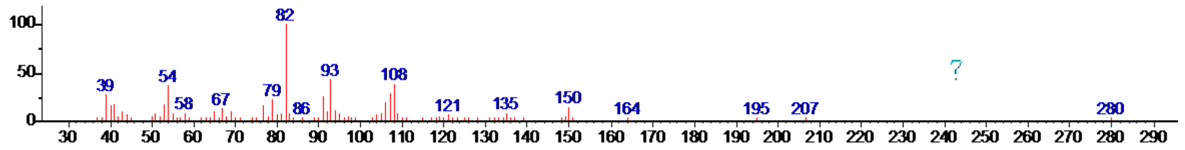

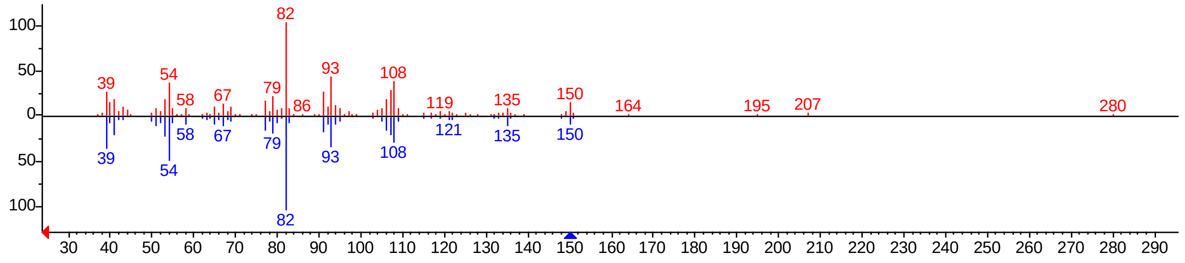

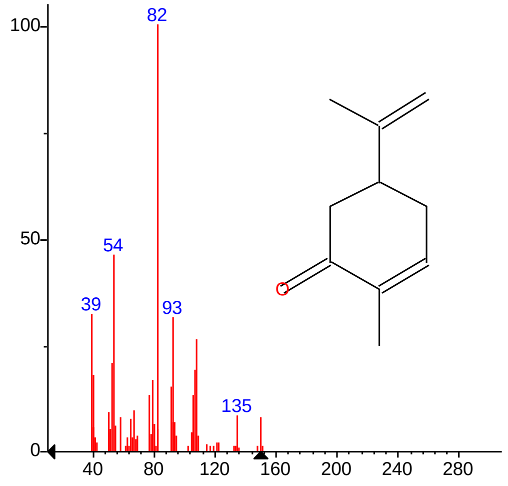

Carvacrol

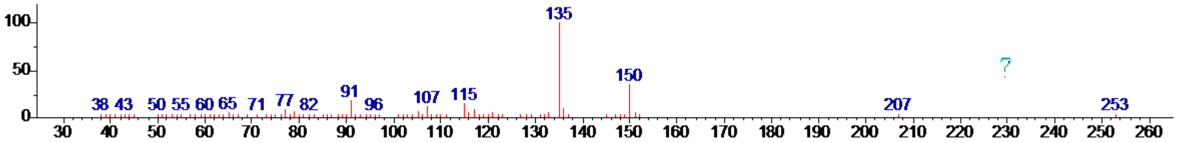

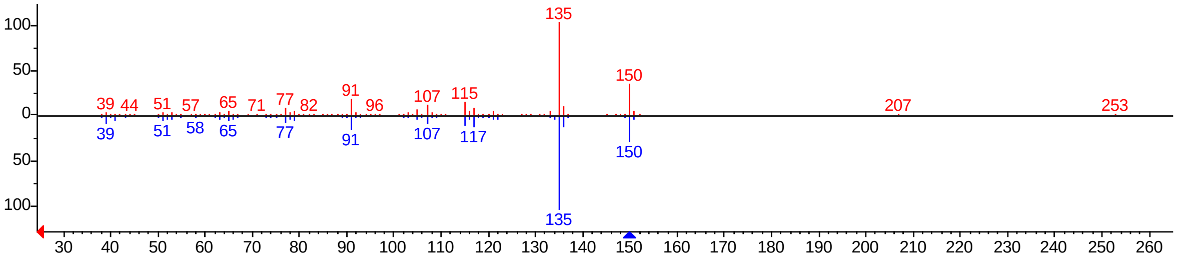

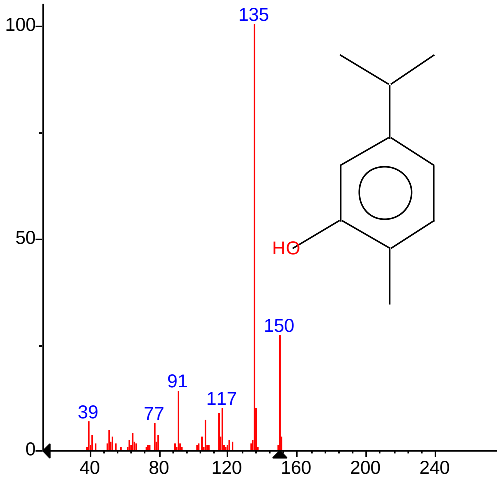

p-Thymol

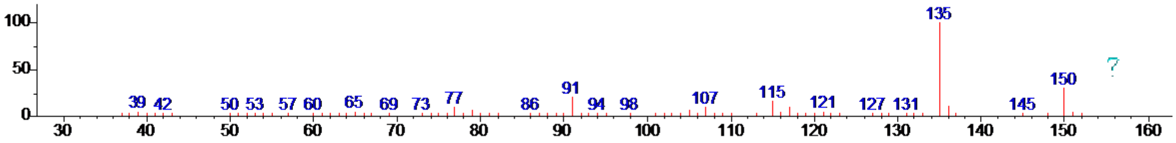

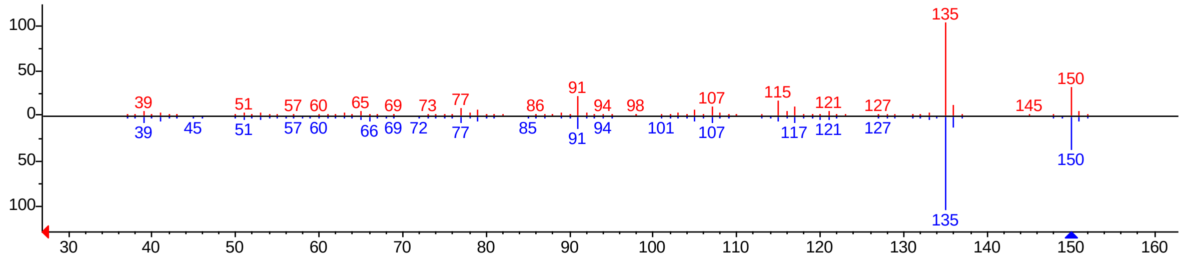
135
150
39 51 57 60 65 69 73 77 86 91 94 98 107 115 121 127 145
39 45 51 57 60 66 69 72 77 85 91 94 101 107 117 121 127
150
135
100 50 0 50 100
30 40 50 60 70 80 90 100 110 120 130 140 150 160

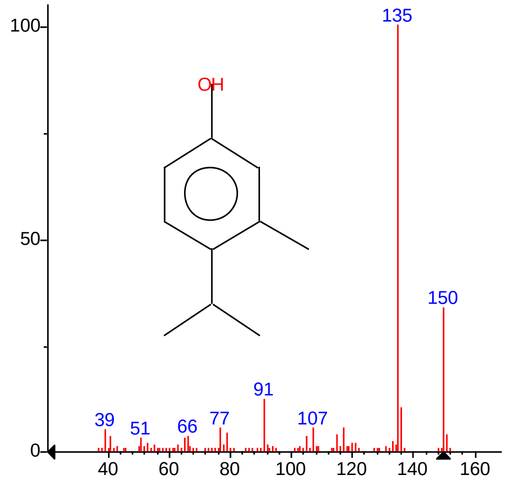
OH
135
150
39 51 66 77 91 107
100 50 0
40 60 80 100 120 140 160

Thymol

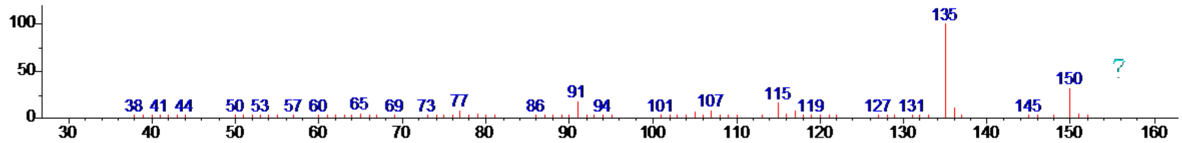
135
150
38 41 44 50 53 57 60 65 69 73 77 86 91 94 101 107 115 119 127 131 145
100 50 0
30 40 50 60 70 80 90 100 110 120 130 140 150 160

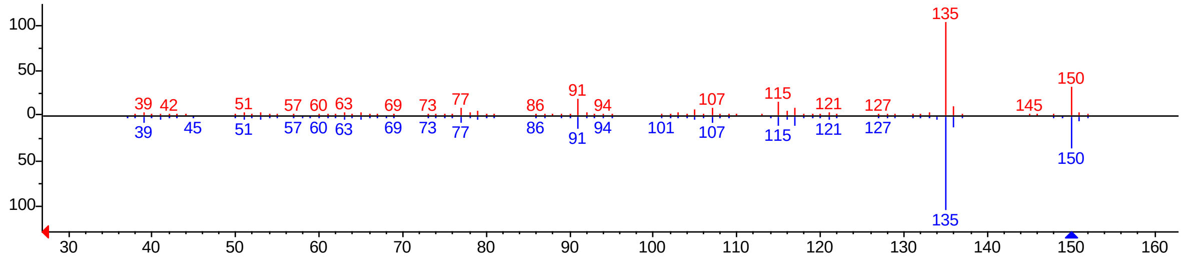
135
150
39 42 51 57 60 63 69 73 77 86 91 94 107 115 121 127 145
39 45 51 57 60 63 69 73 77 86 91 94 101 107 115 121 127
150
135
100 50 0 50 100
30 40 50 60 70 80 90 100 110 120 130 140 150 160

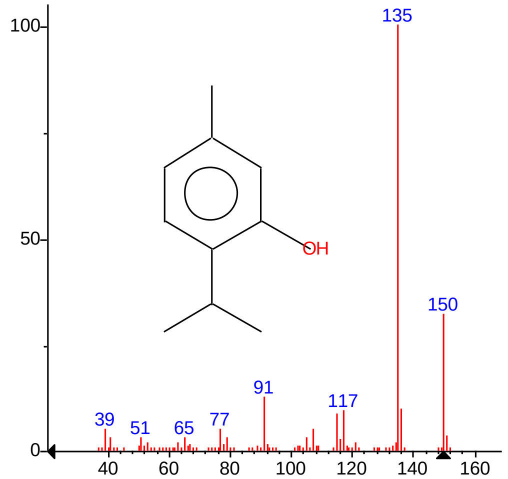

(-)-β-Caryophyllene

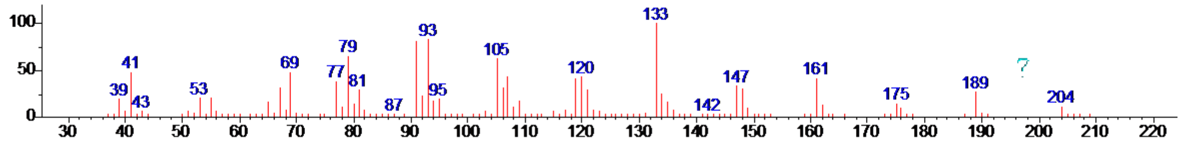

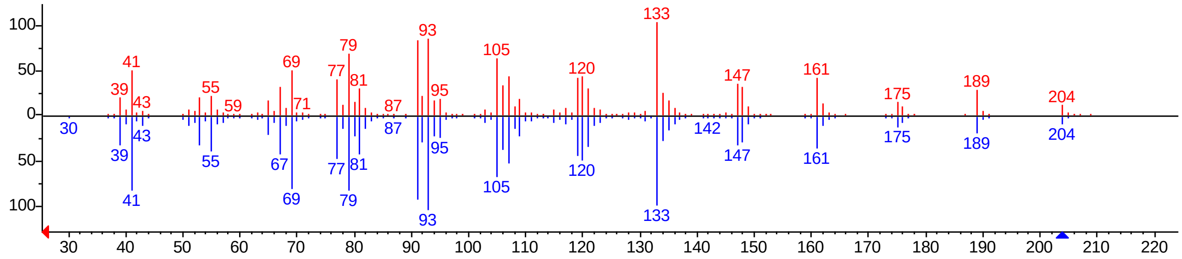

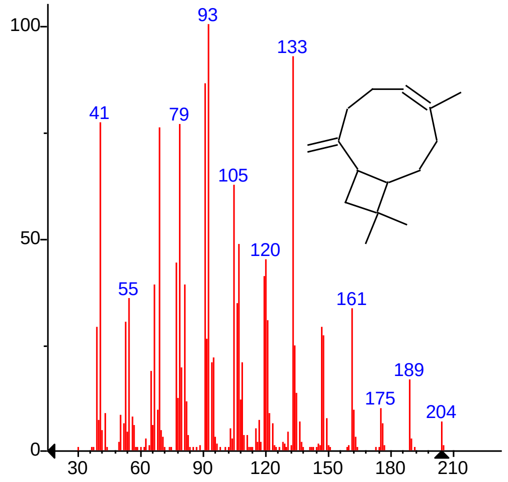

α-Caryophyllene

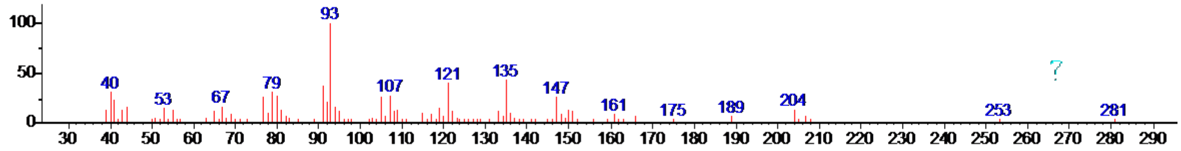

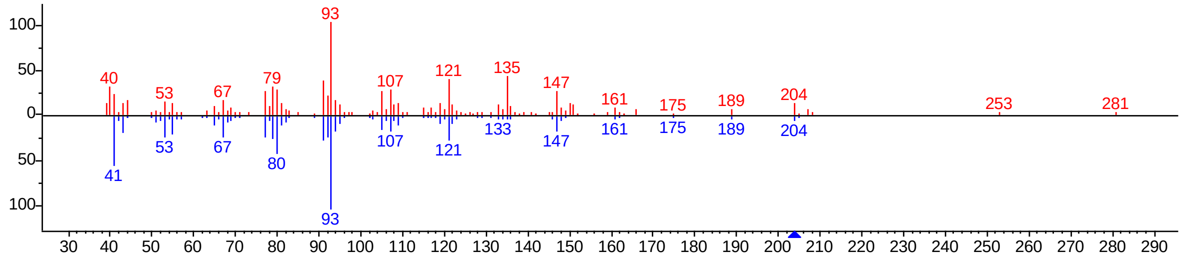

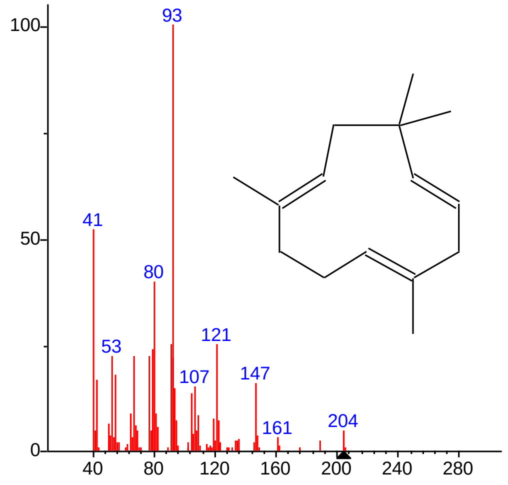

Ƴ-Muurolene

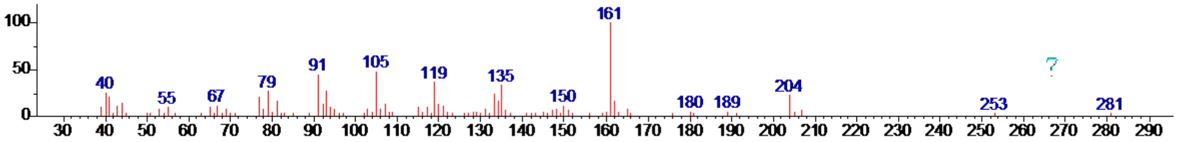

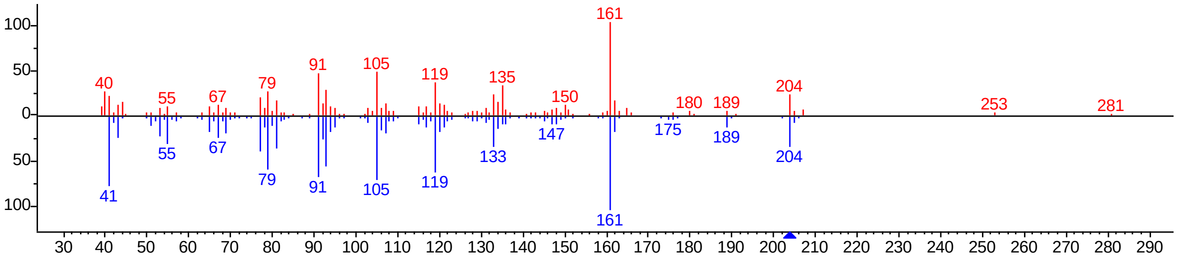

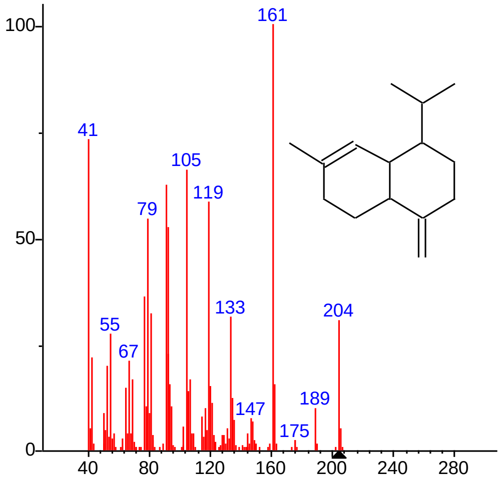

α-Eudesmol

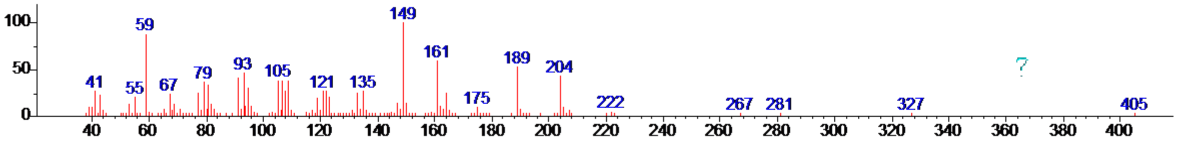

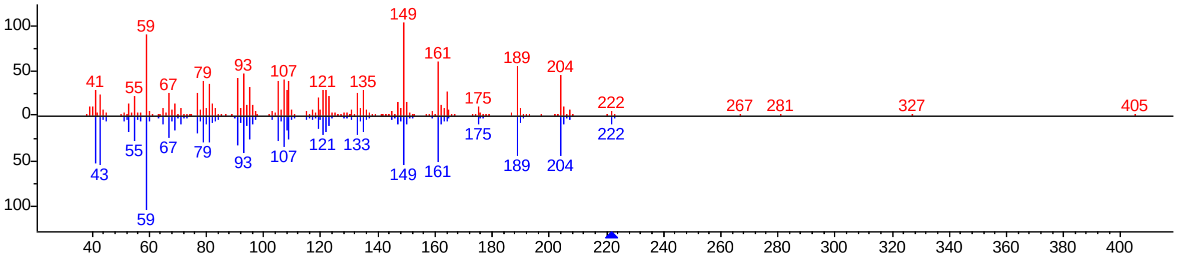

100
50
0
50
100
59
149
161
189
204
41
55
67
79
93
107
121
135
175
222
267
281
327
405
43
55
67
79
93
107
121
133
149
161
175
189
204
222
59
40
60
80
100
120
140
160
180
200
220
240
260
280
300
320
340
360
380
400

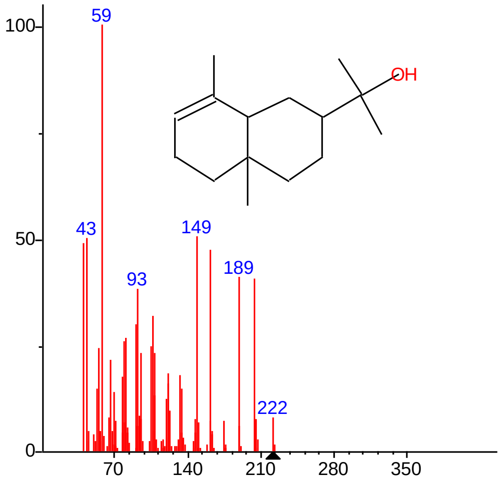

100
50
0
59
43
149
93
189
222
OH
70
140
210
280
350
